# Single‐Atom Iron‐Catalyzed Oxidative Esterification: From Methylarenes to the Upcycling of Polystyrene and Lignin‐Derived Feedstocks

**DOI:** 10.1002/anie.6974259

**Published:** 2026-05-30

**Authors:** Zhuang Ma, Binyu Zhang, Hui Yang, Yue Hu, Junzheng Yu, Yanbin Cui, Jun Zhao, Matthias Beller, Rajenahally V. Jagadeesh

**Affiliations:** ^1^ Leibniz‐Institut Für Katalyse e.V. Rostock Germany; ^2^ Guangzhou Institute of Energy Conversion Chinese Academy of Sciences Guangzhou China; ^3^ National Institute of Clean and Low Carbon Energy Beijing China; ^4^ Department of Biology Hong Kong Baptist University Hong Kong, SAR China; ^5^ Nanotechnology Centre Centre For Energy and Environmental Technologies VŠB‐Technical University of Ostrava Ostrava‐Poruba the Czech Republic

**Keywords:** aromatic esters, atomically dispersed iron catalyst, C–H‐oxidative esterification reaction, polystyrene and plastic, lignin derivatives

## Abstract

The catalytic oxidation of C─H bonds represents a transformative strategy for converting abundant hydrocarbons into high‐value functional molecules. We report a sustainable heterogeneous methodology for the oxidative esterification of methyl (hetero)arenes and the oxidative C─C bond cleavage of alkyl arenes to synthesize aromatic methyl esters using molecular oxygen in water. This process is driven by an atomically dispersed iron catalyst supported on N‐doped porous carbon (Fe‐SAC), featuring well‐defined Fe‐N_4_ active sites. The catalyst exhibits exceptional activity and selectivity, effectively overcoming the intrinsic inertness of C(sp^3^)─H and C─C bonds without the need for noble metals or hazardous additives. Mechanistic investigations, combining kinetic studies, in situ DRIFTS, and DFT simulations, identify superoxide radicals (O_2_^●−^) as key reactive species and pinpoint benzylic C─H bond cleavage as the rate‐determining step. Beyond small molecules, the Fe‐SAC system demonstrates high efficiency in the valorization of lignin derivatives and the chemical upcycling of polystyrene waste into value‐added esters. Its remarkable stability, ease of recycling, and use of an eco‐friendly solvent/oxidant system make this iron‐catalyzed process a cost‐effective and green alternative to traditional esterification methods.

## Graphical abstract

Heterogeneous iron‐catalyzed environmentally benign and sustainable CH‐oxidative esterification methodology has been developed for the synthesis of esters. This challenging reaction has been achieved by the rational design of specific single atom iron‐based catalyst with Fe‐N_4_ active centers, which empowered the oxidative esterification of methyl and alkyl (hetero)arenes as well as lignin‐based feedstocks and polystyrene plastics to produce aromatic esters.

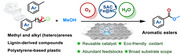

## 1 Introduction

**FIGURE 1:**
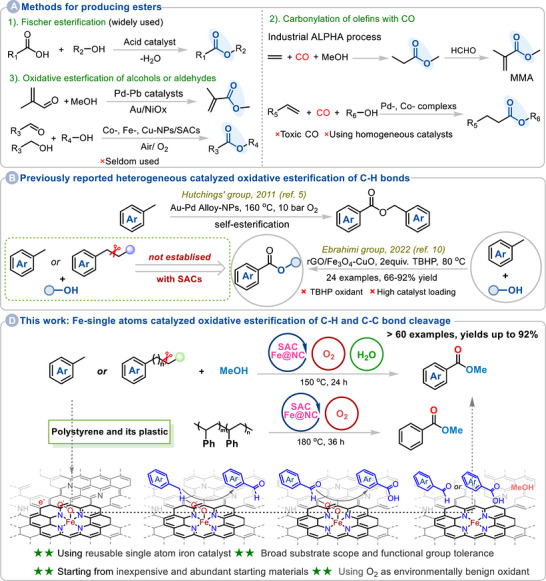
Synthesis of esters. (A) Methods for producing esters. (B) Previously reported heterogeneous catalyzed oxidative esterification of C─H bonds and (C) this work on Fe‐single atoms catalyzed oxidative esterification of C─H and C─C bond cleavage using O_2_.

**FIGURE 2:**
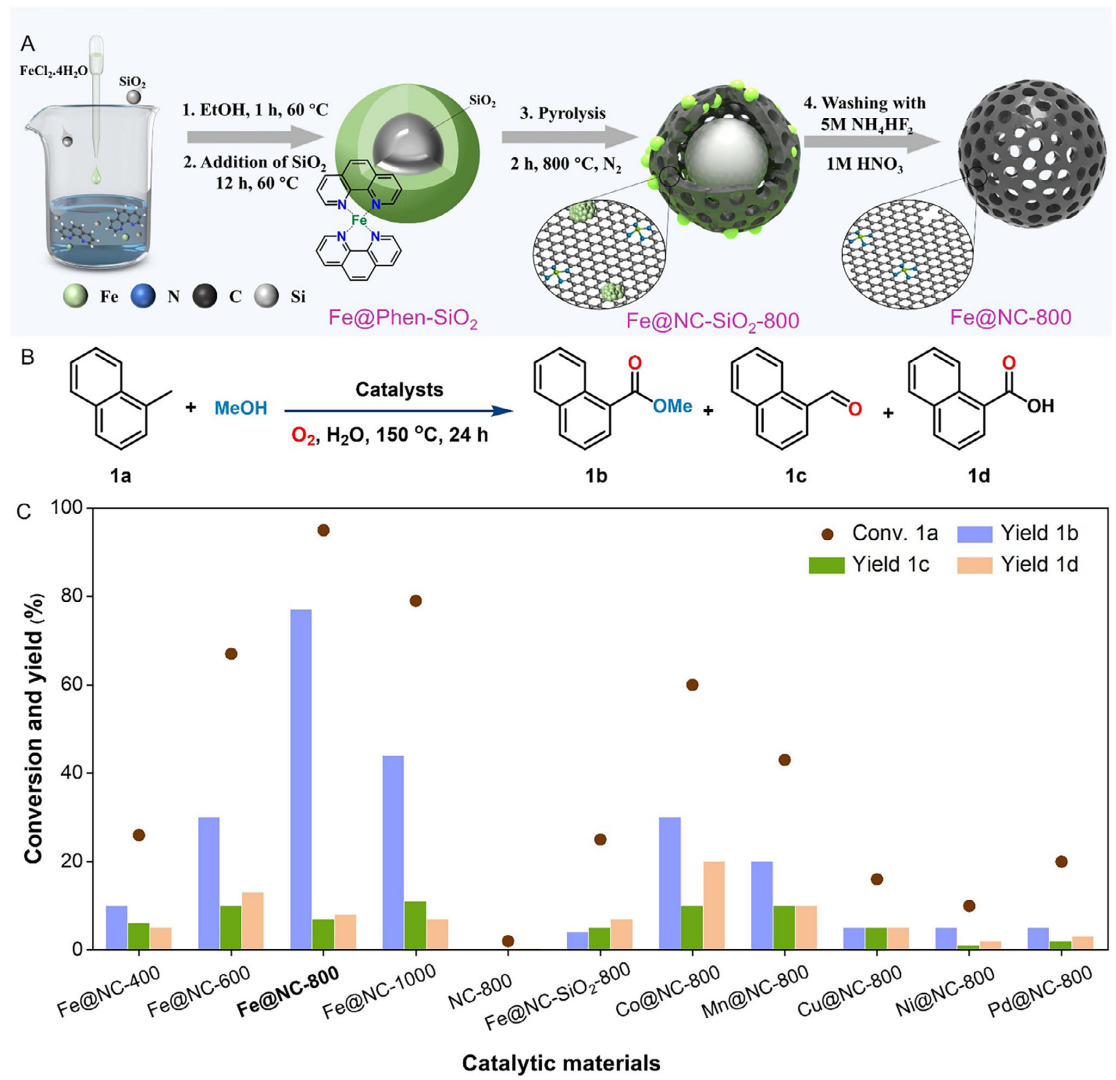
Catalyst preparation and benchmark reaction. (A) Schematic representation of catalyst preparation; (B) Oxidative esterification of 1‐methylnaphthalene to methyl 1‐naphthoate as the model reaction. (C) Catalytic performance of different materials in the model reaction. Reaction conditions for panel (C): 1a: 0.2 mmol, 30 mg catalyst (3.5–4.0 mol% metal basis), 5 bar O_2_, 2.5 mL H_2_O, 0.5 mL MeOH (∼62 equiv. based on 1a), 150°C, 24 h.

**FIGURE 3:**
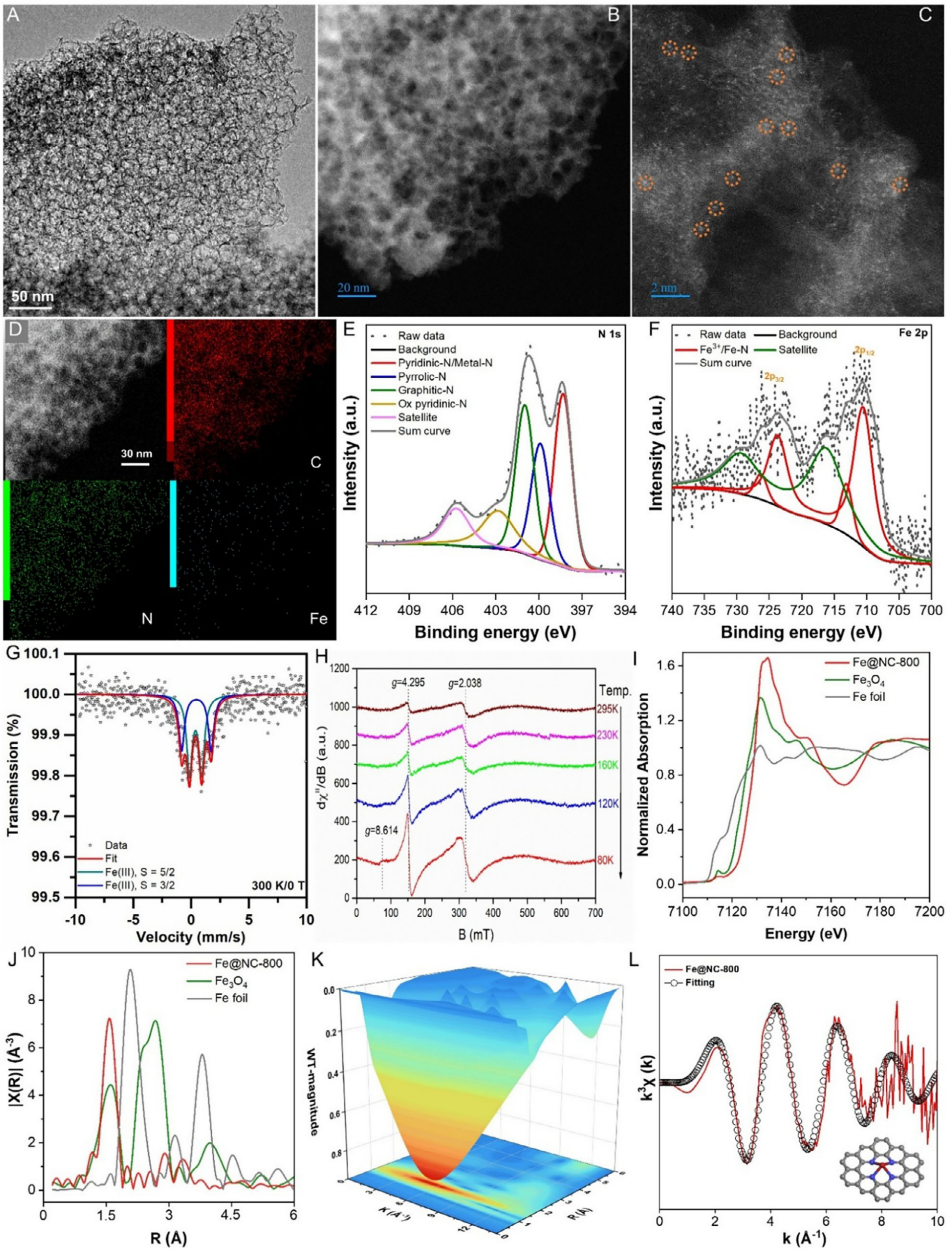
Structure characterizations of the prepared Fe@NC‐800 and references. (A) TEM image, (B, C) HAADF‐STEM images (Fe single atoms are highlighted by origin cycles), (D) EDS mapping images C/N/Fe, (E) N 1*s* and (F) Fe 2*p* x‐ray photoelectron spectroscopy of Fe@NC‐800, (G) ^57^Fe Mössbauer spectra, (H) X‐band CW EPR spectra, (I) normalized XANES at the Fe K‐edge, (J) *k* ^3^‐weighted FT‐EXAFS in R‐space, (K) WT of Fe@NC‐800, and (L) fitting curves in K space.

Hydrocarbons represent one of the most attractive classes of chemical feedstocks due to their high abundance, low cost, and high energy density. The direct C─H functionalization of hydrocarbons is therefore of great importance in modern organic chemistry, offering a cost‐efficient and sustainable route to value‐added chemicals [1, 2, 3, 4, 5, 6]. In particular, the catalytic C(sp^3^)─H oxidation of methyl and alkyl (hetero)arenes with molecular oxygen (O_2_) offers a straightforward and environmentally benign approach for producing a range of synthetically and industrially valuable oxygen‐containing products such as benzylic alcohols, aldehydes, carboxylic acids, esters, and others [5, 6, 7, 8, 9, 10]. Among these, carboxylic acid esters stand out as an essential class of compounds due to their wide applications as fine and bulk chemicals as well as key precursors and intermediates for advanced products, pharmaceuticals, agrochemicals, and polymeric materials [11, 12]. Traditionally, esters are produced by the well‐known and widely used Fischer‐Speier esterification process (Figure 1A–1), in which carboxylic acids react with alcohols in the presence of acid catalysts [13, 14, 15]. This method is extensively applied in both laboratory synthesis and industrial production, particularly when the corresponding carboxylic acids and alcohols are readily accessible. In addition, transition‐metal‐catalyzed carbonylation is also an important platform for ester synthesis, in which olefins are coupled with CO and alcohols to form carboxylic acid esters [16, 17, 18, 19]. A representative industrial example is the ALPHA process, where ethylene, CO, and methanol are converted to methyl propionate as a key intermediate toward methyl methacrylate (MMA) (Figure 1A–2) [16, 17, 18, 19]. Oxidative esterification of alcohols or aldehydes is another ester synthesis route, although its industrial use is limited (Figure 1A–3) [11, 12, 20, 21, 22]. A notable example is the oxidative esterification of methacrolein with MeOH to MMA using Pd/Pb‐ or Au/NiO_x_‐based catalysts [23]. Apart from these processes, the direct CH‐oxidative esterification of readily available hydrocarbons through C─H oxidation and C─C bond cleavage represents an alternative and sustainable approach for the synthesis of esters. Industrial oxidative acetoxylation reactions, such as propylene to allyl acetate and butadiene to 1,4‐diacetoxybutene, also form esters from hydrocarbons [24]. Notably, C─H oxidative esterification of methyl (hetero)arenes using O_2_ or air is considered to be a more practical, step‐economical, and cost‐effective methodology for the synthesis of aromatic esters, as it allows the direct conversion of readily available and inexpensive starting materials. However, this methodology is highly challenging due to the difficulty in the oxidation of inert C(sp^3^)─H bonds in benzylic CH_3_ groups, and hence this reaction mainly relies on precious metal‐based catalysts (Figure 1B). As an example, an Au‐Pd based heterogeneous system has been reported for the oxidative self‐esterification of simple toluenes to the corresponding aromatic esters in the presence of O_2_ [5]. Later, rGO/Fe_3_O_4_‐CuO catalyst was used for the synthesis of aromatic esters starting from methyl aromatic using tert‐butyl hydroperoxide (TBHP) as oxidant and tetra‐n‐butylammonium bromide (TBAB)/tetra‐n‐butylammonium iodide (TBAI) as additive. Obviously, the use of large amounts of TBHP has its inherent disadvantages as an oxidant that limit practical applications (Figure 1B) [10]. To be clear, air or O_2_ is a preferential oxidant for the modern and green oxidation processes due to its availability, non‐toxicity, and it produces only water as the byproduct.

For the advancement of the C─H oxidation process to convert methyl (hetero)arenes to aromatic esters using O_2_ or air, the development and applicability of potential heterogeneous catalytic materials that should be based on more abundant and inexpensive metals, especially iron, is crucial. To increase the activity and selectivity of heterogeneous Fe‐catalysts to accomplish this challenging C─H oxidation reaction, the creation of supported single‐atom iron species is highly desirable due to the advantages of this modern class of materials, which mimic both homogeneous and heterogeneous catalysis aspects with respect to activity and selectivity as well as stability and recyclability [25, 26, 27, 28]. Due to their beneficial aspects and salient features, in recent years, significant research attention has been devoted to the development of single‐atom‐based catalysts (SACs) [29, 30, 31, 32]. As a result, several research groups, including us, have prepared and applied SACs for hydrogenation, animation, oxidation, and other reactions [6, 25, 26, 28, 29, 30, 31, 32, 33, 34, 35]. With respect to C─H oxidation, we and others have reported Fe‐based single atoms for benzylic and allylic C(sp^3^)─H bond oxidation and ammoxidation reactions using O_2_ [6, 28, 35]. Inspired by our previous works, we turned our attention to developing tailored Fe‐SACs for oxidative esterification of methyl and alkyl (hetero)arenes. A key requirement for this transformation is the creation of well‐defined Fe‐N_4_ sites, analogous to those in cytochrome P450 and biomimetic iron complexes, which represent promising catalysts for C─H oxidation processes [36, 37]. Based on this concept, here we report the preparation and application of highly dispersed iron single atoms (Fe‐SAs) supported on N‐doped porous carbon with Fe‐N_4_ centers as a C─H oxidation catalyst (Figure 1C). This Fe‐SAC with Fe‐N active centers was prepared by the generation of silica‐Fe‐nitrogen complex using 1,10‐phenanthroline (Phen) and pyrolysis of this templated material under nitrogen atmosphere followed by the removal of silica and larger particles. The resulting Fe‐SAC demonstrated high stability and reusability as well as sufficient activity and selectivity for oxidative esterification of simple, substituted, and functionalized methyl‐ and heteroarenes with MeOH using O_2_ in water to produce corresponding aromatic methyl esters. In addition to the C─H oxidation of methylated compounds, this Fe‐catalyst has been applied to the oxidative C─C bond cleavage in alkyl (hetero) arenes to prepare methyl benzoates and related esters. Further, the applicability of this Fe‐based C─H‐oxidative esterification is demonstrated for the valorization of lignin‐derived compounds and polystyrene‐based plastics to produce esters.

## 2 Results and Discussion

### 2.1 Preparation and Catalytic Evaluation of Fe‐Based Single Atoms

Over the last decade, we have been focusing on the development of Fe‐based heterogeneous materials for sustainable organic synthesis [6, 25, 26, 33]. Successful implementation of these materials for the desired catalytic application is based on the creation of specific iron‐single atoms supported on N‐doped carbon with Fe‐N active centers. Notably, stability, activity, and selectivity of Fe‐atoms can be controlled by creating an iron‐nitrogen coordination environment. In continuation of our work on SACs, we turned our interest to develop appropriate Fe‐based single‐atom catalyst for C─H‐oxidative esterification reaction. At the initial stage of this work, a series of Fe‐catalysts supported on porous N‐doped carbon were synthesized by the creation and pyrolysis of silica‐Fe‐N complex templated material and subsequent removal of residual silica. Specifically, FeCl_2_ **^.^** 4H_2_O and phenanthroline (Phen) were dissolved in ethanol to form a homogeneous metal‐N complex solution, which was subsequently impregnated onto a silica template generated by LUDOX HS‐40 (40 wt.% aqueous suspension). The resulting SiO_2_‐Fe‐Phen templated material was subjected to pyrolysis under a nitrogen atmosphere at different temperatures (400°C–1000°C) for 2 h. Finally, the pyrolyzed Fe‐material was etched with 5 M ammonium hydrogen difluoride (NH_4_HF_2_) and with 1 M HNO_3_ for removing residual silica and larger particles to obtain the desired Fe‐atoms supported on N‐doped hierarchically porous carbon (Figure 2A). These materials were denoted as Fe@NC‐T, where T represents the pyrolysis temperature.

To find the optimal catalyst, all these Fe‐materials (Fe@NC‐T) have been tested for the oxidative esterification of 1‐methylnaphthalene (**1a**) with MeOH to produce methyl 1‐naphthoate (**1b**) in the presence of O_2_ in water–MeOH (5:1) solvent mixture as a benchmark reaction (Figure 2B). Among different Fe‐materials, the one pyrolyzed at 800°C exhibited more activity and selectivity. With this material, 77% yield of the desired **1b** was obtained with 95% conversion of **1a**. This shows that the formation of optimal catalytic material is based on the applied pyrolysis temperature that creates a desired catalytic structure with Fe‐N centers [25]. Although methylnaphthalene is immiscible in water, the Fe@NC‐800 catalyst, under vigorous stirring, promotes the oxidation/esterification reaction in the presence of MeOH via heterogeneous catalytic conditions at the solid–liquid/liquid–liquid interface. In addition to the oxidative esterification of methylnaphthalene **1a**, we also observed the oxidation of methanol (21% conversion) with the formation of formaldehyde (5%), formic acid (8%), and methyl formate (3%) as byproducts (Table S6). Next, to elucidate the structural factors underpinning catalytic activity, several control materials were evaluated, such as a material with SiO_2_ (without removing silica; Fe@NC‐SiO_2_‐800; Figure 2C) and the one prepared in the absence of nitrogen ligand (Fe@SiO_2_‐800, Table S1, Entry 1); both these exhibited significantly less activity. Similarly, the homogeneous iron complex (FeCl_2_ + Phen, Table S1, Entry 2) also displayed inferior activity, highlighting the essential role of heterogeneous architecture with Fe atoms supported on N‐doped carbon. Next to compare the activity of Fe@NC‐800 catalyst, other metal‐base catalysts such as Mn@NC‐800, Co@NC‐800, Cu@NC‐800, and Ni@NC‐800 were prepared in a manner similar to that of Fe‐materials (Figure 2C). All these materials exhibited less activity and selectivity to that of Fe@NC‐800. Furthermore, a selection of commercially available catalysts, including iron phthalocyanine (FePC), Pd/C, Ru/C, Rh/C, Pt/C, and nano CuO were tested in the model reaction. These results confirmed that Fe@NC‐800 was found to be more active and selective compared to all these commercial catalysts (Table S1, Entries 3‐9). To further examine the effect of Fe loading, Fe@NC‐800 catalysts with different Fe contents were prepared by varying the amount of FeCl_2_·4H_2_O precursor. The performance of these Fe materials demonstrated a clear dependence on the Fe loading, with the optimized Fe@NC‐800 giving the highest yield of the desired product, whereas ones with lower or higher Fe loading led to decreased catalytic activity (Table S1, Entries 10–13). This trend is likely attributable to the insufficient formation of Fe‐N_4_ centers at lower Fe loading, while at high Fe loading, less efficient Fe dispersion or possible Fe aggregation occurs. In addition, Fe@NC‐800‐7 nm, prepared using AEROSIL 300 fumed silica (∼7 nm) instead of colloidal silica (LUDOX AS‐40), gave a lower yield of the desired product, suggesting that the SiO_2_ template particle size and morphology also affect the catalyst structure and performance (Table S1, Entry 14). Normally, the solvent plays a key role in the performance of any chemical reaction. Therefore, the model reaction was tested in different solvents, and this C‐H oxidation rection proceeded well in water compared to other tested solvents (Table S3). Further, the benchmark reaction was tested at temperature with different pressures of molecular oxygen (Table S4). All results confirmed that the best result with 77% yield of **1b** and TOF of 0.9 h^−1^ (Table S5) was obtained at 150°C with 5 bar O_2_ using 30 mg Fe@NC‐800 (3.55 mol% Fe) in water as the solvent.

### 2.2 Structural Characterization of Fe‐Based Catalytic Materials

To understand the structural features and catalytic performance of the prepared iron catalysts and to establish a structure‐activity relationship, the optimal fresh catalyst (Fe@NC‐800) and the recycled one (Fe@NC‐800‐R) were characterized using inductively coupled plasma optical emission spectroscopy (ICP‐OES), x‐ray powder diffraction (XRD), Raman spectroscopy, oxygen temperature programming desorption (O_2_‐TPD), transmission electron microscopy (TEM), scanning electron microscopy (SEM), Brunauer‐Emmet‐Teller (BET), high‐angle annular dark‐field STEM (HAADF‐STEM), energy dispersive x‐ray spectroscopy (EDS), x‐ray photoelectron spectroscopy (XPS), ^57^Fe Mössbauer spectroscopy, electron paramagnetic resonance (EPR) spectroscopy, extended x‐ray absorption fine structure (EXAFS), and x‐ray absorption near edge structure spectroscopy (XANES) data collection.

First, the content of Fe in the fresh and recycled catalysts was quantified by ICP‐OES, and it was found that both materials contained almost the same amount of Fe (Fe@NC‐800 = 1.32 wt% of Fe; Fe@NC‐800‐R = 1.31 wt% Fe). Next, the XRD patterns of both materials are quite similar and indicate the dominant presence of two distinct diffraction peaks around 25° and 44°, which show similarities to the (002) and (101) crystal planes of graphitic carbon (Figure S2) [19]. Notably, no diffraction signals associated with crystalline metallic iron or iron oxides were detected, indicating that the iron species are highly dispersed within the carbon matrix. Then, Raman spectroscopy provided further structural insights, displaying two distinct peaks at 1342 and 1582 cm^−1^ (Figure S3), which are assigned to the disordered carbon (D band) and the crystalline graphitic carbon (G band, *I_D_/I_G_* is 1.004), respectively. In general, the relative intensity ratio of D bands to G bands (*I_D_/I_G_*) can be used to estimate the defective degree of carbon materials. Next, the distinct desorption peak at around 110 °C in the O_2_‐TPD profile indicates a high affinity of Fe@NC‐800 towards oxygen adsorption (Figure S4). This can be attributed to the presence of abundant Fe‐N_x_ sites and porous carbon structures that facilitate the physisorption or weak chemisorption of O_2_ at low temperatures [38, 39]. In addition to the low‐temperature feature, broad desorption signals observed in the intermediate range (200°C–400°C) are assigned to the release of chemistry‐adsorbed O_2_ in the sample, which are typically involved in surface redox processes [38, 39].

Both TEM and SEM analysis revealed that the well‐defined mesoporous N‐doped carbon structure and absence of nanoparticles, in line with the XRD findings (Figures 3A,B, S6, and S7). Evidently, the mesoporous architecture is formed through the removal of SiO_2_, resulting in a large specific BET surface area (827.3 m^2^/g) as well as a mesoporous structure (pore diameter 10.6 nm) of Fe@NC‐800 catalyst, as confirmed by N_2_ adsorption‐desorption isotherms and pore size analyses. However, the silica‐containing material Fe@NC‐SiO_2_‐800 exhibited a low specific surface area (12.3 m^2^/g) and a small pore diameter (3.6 nm) (Figure S5 and Table S6). These results clearly highlight the critical role of silica‐template removal in constructing a porous carbon framework. Such a hierarchical mesoporous structure can facilitate the diffusion and transport of substrates and O_2_, improve accessibility to atomically dispersed Fe‐N_4_ sites, and thereby contribute synergistically to the enhanced catalytic performance of Fe@NC‐800 [33, 34]. Simultaneously, HAADF‐STEM images (Figures 3C and S8) showed well‐dispersed Fe‐species (light dots) anchored on the entire carbon architecture. Owing to the significant difference in Z‐contrast between light elements (C, N) and heavier atoms such as Fe, these light dots were identified to be Fe atoms and marked by orange circles. At the same time, the atomic dispersion of the Fe species was also confirmed by corresponding EDS analysis (Figure S9). Moreover, element mapping of Fe@NC‐800 demonstrates a uniform spatial distribution of Fe, N, and C elements throughout the material (Figure 3D), corroborating the successful incorporation of Fe atoms into the N‐doped carbon matrix.

Next, XPS survey spectra (Figure S10) reveal the presence of distinct peaks corresponding to C, N, O, Fe, and a minor Si signal. The Si is in small amounts remaining from the catalyst preparation process. The elemental quantification in Table S7 shows that both catalysts have a Fe surface concentration of 0.2 at.% with nitrogen content of 9.2 and 8.7 at.% for Fe@NC‐800 and Fe@NC‐800‐R, respectively. The N 1*s* spectra in Figures 3E and S12c of both fresh and recycled materials were deconvoluted into five different peaks at binding energies of 398.3, 399.9, 401.0, 402.8, 406.3 eV corresponding to pyridinic, pyrrolic, graphitic, and oxidized pyridinic N species and satellite peak, respectively [40]. In the Fe 2*p* region (Figure 3F) of the fresh catalyst Fe@NC‐800 the main peaks at 710.0 and 723.2 eV (corresponding to Fe 2p_3/2_ and Fe 2p_1/2_) together with pronounced satellite features around 716.3 and 729.5 eV are observed, which indicate the presence of Fe^3+^ or Fe‐N species as the main oxidation state [41]. Interestingly, for the recycled catalyst, Fe@NC‐800‐R, identical iron species are observed with similar binding energies, thus suggesting no changes in the oxidation state compared to the fresh catalyst (Figure S12D).

To get a deeper insight into the chemical nature and local surroundings of probed atoms, ^57^Fe Mössbauer spectroscopy was employed. The room temperature ^57^Fe Mössbauer spectrum of the Fe@NC‐800 sample shows two doublet components (Figure 3G). Based on the derived hyperfine parameters, the doublet with the lower value of the quadrupole splitting parameter is attributed to Fe^3+^ ions in a high‐spin state (*S* = 5/2) [25]. The other doublet with a slightly increased isomer shift value and significantly higher quadrupole splitting parameter reflects the distortion and high asymmetry of the local surroundings of probed Fe atoms and can be assigned to Fe^3+^ ions in the intermediate spin‐state of *S* = 3/2. The observed Mössbauer data are in high agreement with EPR measurements performed on the Fe@NC‐800 sample at different temperatures (Figure 3H). EPR spectra confirmed the presence of two types of iron metal cations in a 3+ oxidation state. The more significant peak arising at 150 mT, which corresponds to the *g*‐value of 4.295, is clearly assignable to Fe^3+^ with *S* = 5/2 (Kramers doublet with m_s_ = <±3/2>). Besides that, the central doublet peak located around 320 mT corresponding to the *g*‐values of about 2.038 can be clearly recognized. This resonant line belongs to Fe^3+^ ions in the spin state of *S* = 3/2 (the m_s_ = <±1/2>) adopting pseudo‐octahedral/tetragonally distorted environment. In summary, two types of Fe^3+^ cations were clearly recognized in the Fe@NC‐800 sample by a combination of Mössbauer and EPR spectroscopies; the dominant fraction corresponds to the spin‐state of *S* = 5/2 with octahedral/rhombic coordination, and the other fraction corresponds to the spin state of 3/2 with pseudo‐octahedral/tetragonally distorted environment.

Subsequently, to verify the bonding state and local coordination structure of iron atoms in Fe@NC‐800, Fe K‐edge synchrotron radiation‐based x‐ray absorption near‐edge structure (XANES) measurements were performed with Fe foil and Fe_3_O_4_ as reference samples. The absorption edge of Fe@NC‐800 is higher than the Fe foil and Fe_3_O_4_ (Figure 3I), indicating that the Fe atom carries a positive charge and the valence state of Fe is more than +2, which is in line with the XPS result. The Fourier‐transformed (FT) *k^3^*‐weighted EXAFS spectra (Figure 3J) displayed one main peak at 1.4 Å, corresponding to the Fe‐N first coordination shell, and no Fe‐Fe peak, typically at 2.2 Å, shows up, ruling out the possibility of the aggregation of anchored Fe atoms. The wavelet transforms (WT) plot (Figures 3K and S13) of Fe@NC‐800 showed the WT maximum at 4.5 Å^−1^ in R space and 1.4 Å^−1^ in K space, respectively, which can be assigned to Fe‐N scattering path. Furthermore, EXAFS fittings confirm that the coordination number of 3.8 for the Fe centers and the mean Fe─N bond length of 2.01 Å (Figure 3L and Table S8). All these structural analyses proved the formation isolated iron single atoms supported on N‐doped mesoporous carbon with Fe‐N_4_ centers in the optimal catalyst.

### 2.3 Applicability of Fe@NC‐800 for CH‐Oxidative Esterification of Methyl (Hetero) Arenes to Esters

**FIGURE 4:**
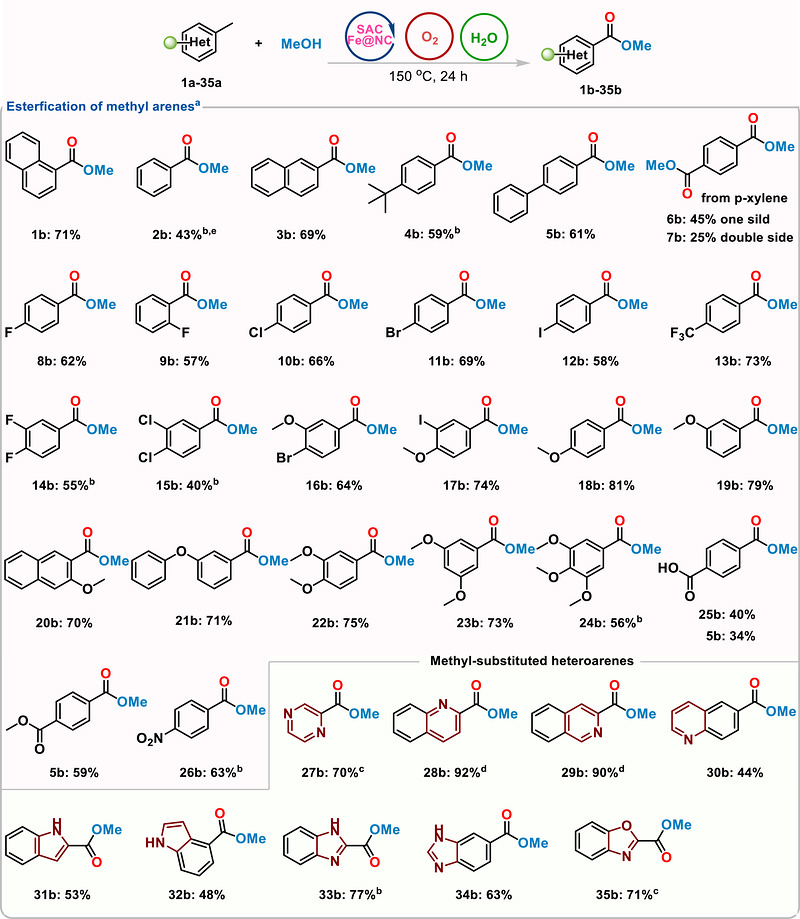
Fe@NC‐800 catalyzed oxidative esterification of different methyl (hetero)arenes. Reaction condition^a^: 0.2 mmol methylarene, 30 mg Fe@NC‐800 (3.55 mol% Fe), 5 bar O_2_, 2.5 mL H_2_O, 0.5 mL MeOH (∼ 62 equiv. based on substrates), 150°C, 24 h, isolated yield. ^b^: Same as “a” with 50 mg Fe@NC‐800 (5.93 mol% Fe) at 160°C. ^c^: Same as “a” with 130°C. ^d^: Same as “a” with 110°C. ^e^: GC yield.

After having designed the desired catalyst and optimal conditions for the model reaction, we performed the C—H‐oxidative esterification of methylarenes and heteroarenes with MeOH to prepare corresponding aryl methyl esters using Fe@NC‐800. First, several simple and substituted toluenes were esterified with MeOH to give corresponding methyl benzoates in good yields (Figure 4; products **1b–5b**). It should be noted that, due to the limited solubility of certain substrates in the H_2_O/MeOH solvent mixture, the oxidative esterification reaction proceeds as a heterogeneous multiphase system under vigorous stirring. Methyl groups in xylenes underwent oxidative esterification to give corresponding mono and diesters (products **6b** and **7b)**. Among these products, the diester, dimethyl terephthalate, is a key precursor for the production of a PET‐based polymer [42]. Halogen‐substituted substrates (F‐, Cl‐, Br‐, I‐, and CF_3_‐) gave the corresponding halogenated methyl benzoates (products **8b–17b**), which serve as valuable building blocks in organic synthesis and drug discovery. Electron‐rich substrates bearing methoxy groups reacted well and produced desired esters in yields up to 75% (products **16b–24b**). In addition, multi‐substituted methylarenes underwent esterification and gave moderate yields of the corresponding esters (products **14b–17b, 22–24b**). Interestingly, when the 4‐toluic acid was subjected to oxidative esterification, apart from monoester **25b** (40%), the formation of diester **5b** (34%) was also observed. The electron‐poor nitro‐substituted toluene was also converted to nitro‐methyl benzoate (product **26b**). In addition, methyl‐heteroarenes were also esterified to produce corresponding heterocyclic esters. As an example, methyl‐pyridine, ‐quinoline, ‐isoquinoline, ‐quinoxaline, ‐indole, ‐imidazole‐, and ‐thiazole gave corresponding heterocyclic methyl esters in good to excellent yields under relatively mild conditions. These esters find applications in the preparation of pharmaceuticals, agrochemicals, and biomolecules (products **27b–35b**).

### 2.4 Fe@NC‐800 Catalyzed Esterification of Alkyl Arenes to Esters via C─C Bond Cleavage

In addition to methylarenes, alkylarenes are also prevalent in nature and can also be generated from lignin biomass as renewable compounds [43]. However, these substrates are less explored as starting materials for making esters. Hence, we became interested in oxidizing different alkylarenes with Fe@NC‐800 in the presence of MeOH to produce corresponding aryl methyl esters. In case of alkylarenes, oxidation at the benzylic C‐H position cooccurred, followed by C─C bond cleavage and subsequent esterification with MeOH to give methyl benzoates.

**FIGURE 5:**
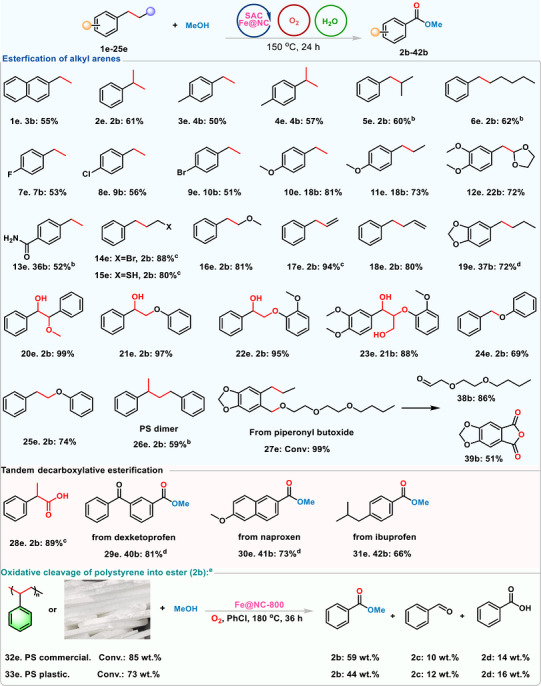
Fe@NC‐800 catalyzed cleavage esterification of different alkylarenes. Reaction condition^a^: 0.2 mmol alkylarene, 30 mg Fe@NC‐800 (3.55 mol% Fe), 5 bar O_2_, 2.5 mL H_2_O, 0.5 mL MeOH (∼ 62 equiv. based on substrates), 150°C, 24 h, GC yield. ^b^: Same as “a” with 160°C, 40 mg Fe@NC‐800 (4.73 mol% Fe). ^c^: Same as “a” with 130°C. ^d^: Isolated yield. ^e^: 2 mmol polystyrene (208 mg), Fe@NC‐800 (6.0 mol% Fe), 10 bar O_2_, 10 mL PhCl, 3 mL MeOH (∼ 37 equiv. based on PS), 180°C, 36 h, GC yield.

As shown in Figure 5, several simple alkylarenes could be cleaved to give the desired aromatic esters in moderate to good yields (substrates **1e–6e**). Similar to methylarenes, F‐, Cl‐, Br‐ and OMe‐ substituted alky benzenes were also converted to corresponding aryl methyl esters also prepared via cleavage of C─C bond (substrates **7e–9e**). Like methylarenes, methoxy‐substituted substrates are more easily transformed into corresponding esters (substrates **10e–12e**). Additionally, selective C‐C cleavage at the benzylic position took place not only in substituted arenes, but also in substrates with functionalization on the alkyl group, such as (3‐bromopropyl)benzene (**14e**), 3‐phenylpropanethiol (**15e**), (2‐methoxyethyl)benzene (**16e**) and allylbenzene (**17e**) as well as 4‐phenyl‐1‐butene (**18e**).

### 2.5 Oxidative Valorization of Lignin Derived Compounds and Polystyrene Plastics to Aromatic Esters

Additionally, the application of this Fe‐catalyzed protocol was demonstrated for the oxidative esterification lignin derived model compounds, which represent renewable feedstocks. To our delight the tested lignin model compounds were all smoothly underwent oxidative cleavage followed by esterification with MeOH to provide excellent aromatic esters in excellent yields (substrates **20e–25e**). Using our Fe‐SAC catalyst, bioactive molecules can also be oxidized and esterified with MeOH. In the case of piperonyl butoxide both C─C and C─O bond cleavage took place to produce the corresponding ether **38b** and anhydrides **39b** (substrate **27e**). Subsequently, we conducted the oxidative esterification of drug molecules, dexketoprofene‐, naproxen‐, and ibuprofen‐containing methyl propionic acid groups (substrates **28e–31e**). In these cases, oxidative decarboxylation and esterification took place to give corresponding aromatic methyl esters. Next, to show case the viability of this Fe‐based oxidative esterification was tested for the conversion of polymeric materials and plastic waste. First, polystyrene dimer substrates were oxidized in presence of MeOH and generated methyl benzoate (**2b**) in 36% yield (substrate **26e**). We next examined the oxidative esterification of polystyrene (PS)—a widely used yet chemically resistant polymer composed of inert C─C linkages and aromatic units [44, 45]. Noteworthy, this polymeric material was decomposed with C─C bond cleavage and esterification to provide methyl benzoate. Chemical recycling of PS remains a long‐standing challenge owing to its thermal stability and non‐polar backbone. Remarkably, our Fe@NC‐800 catalyst enabled the direct oxidative cleavage of both commercial (substrate **32e**) and post‐consumer (substrate **33e**) PS, affording methyl benzoate (**2b**) as the major product, along with benzaldehyde (**2c**) and benzoic acid (**2d**) in minor yields.

### 2.6 Demonstrating Stability, Recycling, and Reusability of Fe@NC‐800 Catalyst

**FIGURE 6:**
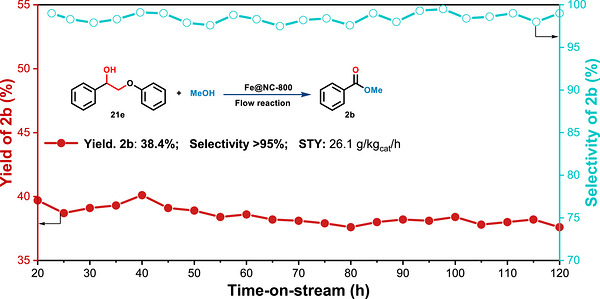
Long‐term stability of Fe@NC‐800 catalyst. The reaction procedure and conditions are placed in Supporting Information. WHSV = the weight of feed per hour/the weight of the catalyst; STY = amount of product (g)/catalyst (kg)/reaction time (h).

In general, stability, recycling and reusability are essential factors to validity the successful implantation of any heterogeneous catalysts. In this regard, we demonstrated these aspects of our catalyst, Fe@NC‐800 for this challenging C─H‐oxidative esterification. First, to assess the heterogeneous nature of the Fe@NC‐800 catalyst, a hot filtration experiment was conducted after the reaction has been run for 12 h with a conversion of **1a** reached to approximately 50%. Following the filtration, the oxidative esterification rection was performed again using the filtrate under identical conditions. The reaction did not occur in the filtrate, which indicates this C─H oxidation process proceeds via heterogeneous catalysis conditions (Figure S1A, green curve). Additionally, analysis of the filtrate by ICP‐OES revealed the absence of iron species, confirming that Fe@NC‐800 operates as a true heterogeneous catalyst without any leaching of iron species into the solution. Subsequently, the recycling and reusability performances of Fe@NC‐800 were evaluated by conducting the model reaction for 24 and 12 h. As shown in Figure S1B, Fe@NC‐800 exhibited high stability and was recycled and reused up to 6 times without significant loss of its catalytic performance. Characterization of the recycled catalyst (Fe@NC‐800‐R; Figures S2, S10, and S12; Table S6) further supports these results, showing that the catalyst retains structural integrity and high efficiency even after multiple reuse cycles. These results underline the robust stability and reusability of Fe@NC‐800, making it a promising catalyst for advancing this CH–oxidation process. Apart from the batch recycling experiments, we also tested the long‐term stability Fe@NC‐800 by continuous flow reaction. For this purpose, we chose the β‐O‐4 lignin model compound **21e,** which was subjected to the oxidative esterification reaction with MeOH in a flow reactor. As shown in Figure 6, the catalyst maintained its stability and performance for 120 h without any decrease in the yield of methyl benzoate (**2b**). During the whole process, the overall weight hourly space velocity (WHSV) and space‐time yield (STY) is 0.129 h^−1^ and 26.1 g/kg_cat_/h, respectively.

### 2.7 Mechanistic Investigations for the Fe@NC‐800 Catalyzed CH–Oxidative Esterification

**FIGURE 7:**
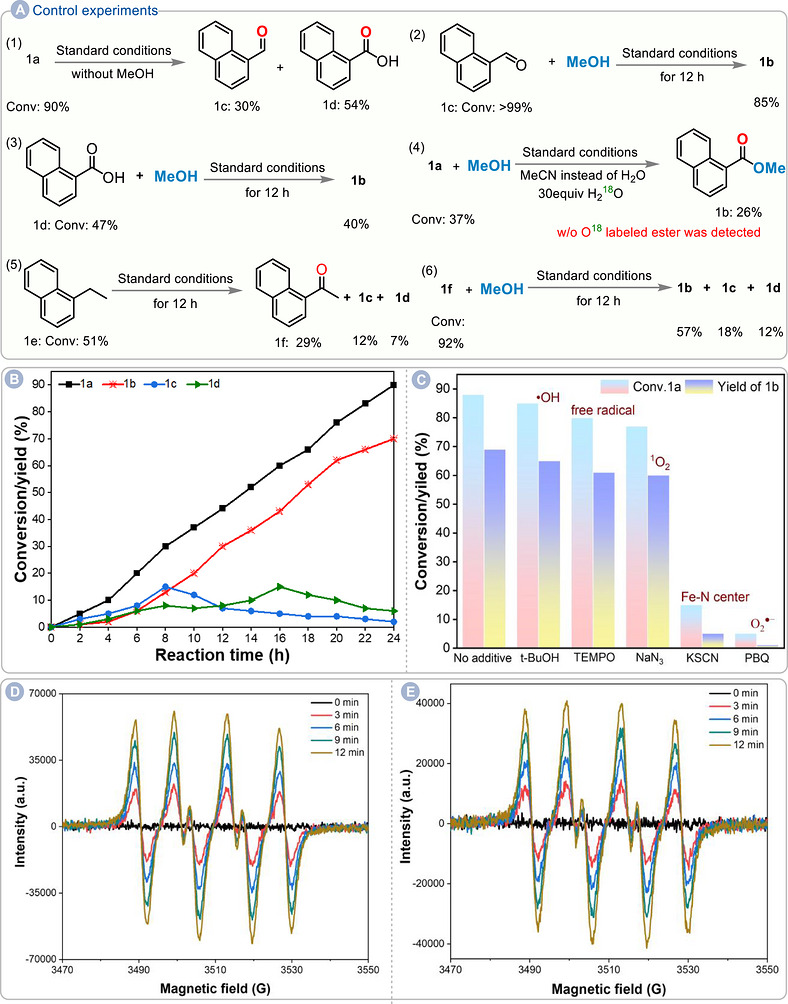
Mechanism insight of esterification of methylarenes. (A) Control experiments. (B) Time course profile. (C) Radical trapping experiments with different additives. DMPO spin‐trapping EPR spectrum of O_2_ with (D) Fe@NC‐800 and (E) Fe@NC‐SiO_2_‐800 in MeOH.

Subsequently, to gain insight into the possible reaction pathway, we carried out control experiments, reaction kinetic, and in situ spectroscopic study. As shown in Figure 7A–1, **1a** was first converted to the corresponding aldehyde (**1c**, 30%) and carbonylic acid (**1d**, 54%) in the absence of MeOH under the standard conditions. Hence, we postulate that **1c** and **1d** are reaction intermediates involved in this CH‐oxidative transformation. Subsequently, the control experiments started from **1c, 1d,** as well as reaction kinetic curves were collected, respectively. Clearly, **1b** was successfully prepared from **1c** or **1d** with a yield of 85% and 40%, respectively, in the presence of MeOH (Figure 7A–2,A‐3). Similarly, the kinetic curves also show the presence of **1c** and **1d** increasing first and then decreasing (Figure 7B), suggesting that the reaction may involve two pathways. One path being the direct oxidative esterification of aldehydes with MeOH via the formation of hemiacetal, and the other one is the Fischer esterification of carboxylic acid and MeOH. Additionally, to further explore the role of H_2_O and O_2_ in this reaction, next we conducted an isotope‐labeling experiment with H_2_^18^O or O_2_ as an oxygen source, respectively. These results showed that no ^18^O‐labeled ester was detected after reaction, which revealed that oxygen atoms of ester originated from O_2_ rather than H_2_O (Figure 7A–4) [33]. Then, radical quenchers/trapping experiments were conducted to identify reactive oxygen species (ROS) in the presence of different radical scavengers. The addition of *tert*‐BuOH or NaN_3_ as scavengers for quenching singlet oxygen (^1^O_2_), hydroxyl (^●^OH) radicals, respectively, did not affect the reaction efficiency, which ruled out the involvement of these types of radicals in the reaction system (Figure 7C, Columns 2 and 4). Similarly, adding 2,2,6,6‐tetramethyl‐1‐piperidinyloxyl (TEMPO) into the reaction system for quenching any involved free radicals did not affect the yield of **1b** (Figure 7C, Column 3), again suggesting the non‐radical nature of the overall process. However, the reaction is inhibited after the addition of 1,4‐benzoquinone (PBQ), which is a reagent for quenching superoxide (O_2_^●−^) species (Figure 7C, Column 6). All these experiments indicated that O_2_^●−^ is formed during the reaction. Furthermore, this assumption is supported by the in situ EPR experiments in the presence of 5,5‐dimethyl‐1‐pyrroline N‐oxide (DMPO) as a spin trap agent, a spin signal with the intensity ratio of 1:1:1:1:1:1 was observed, which is assigned to the signal of DMPO‐O_2_^●−^ adduct and the intensity increased with time (Figure 7D) [6]. For comparison, the signal intensity of O_2_^●−^ arising from Fe@NC‐SiO_2_‐800 is obviously weaker than that of Fe@NC‐800 (Figure 7E). Next, to verify the catalytic role of Fe‐N sites, a poisoning experiment using potassium thiocyanate (KSCN) was performed. It has been shown that the addition of SCN^−^ could effectively block and deactivate active metal‐N‐C catalysts. Indeed, a significant decrease in the activity was observed in the presence of KSCN (Figure 7C, Column 5), suggesting the involvement of atomic Fe‐N sites in this catalytic CH‐oxidation process [28].

**FIGURE 8:**
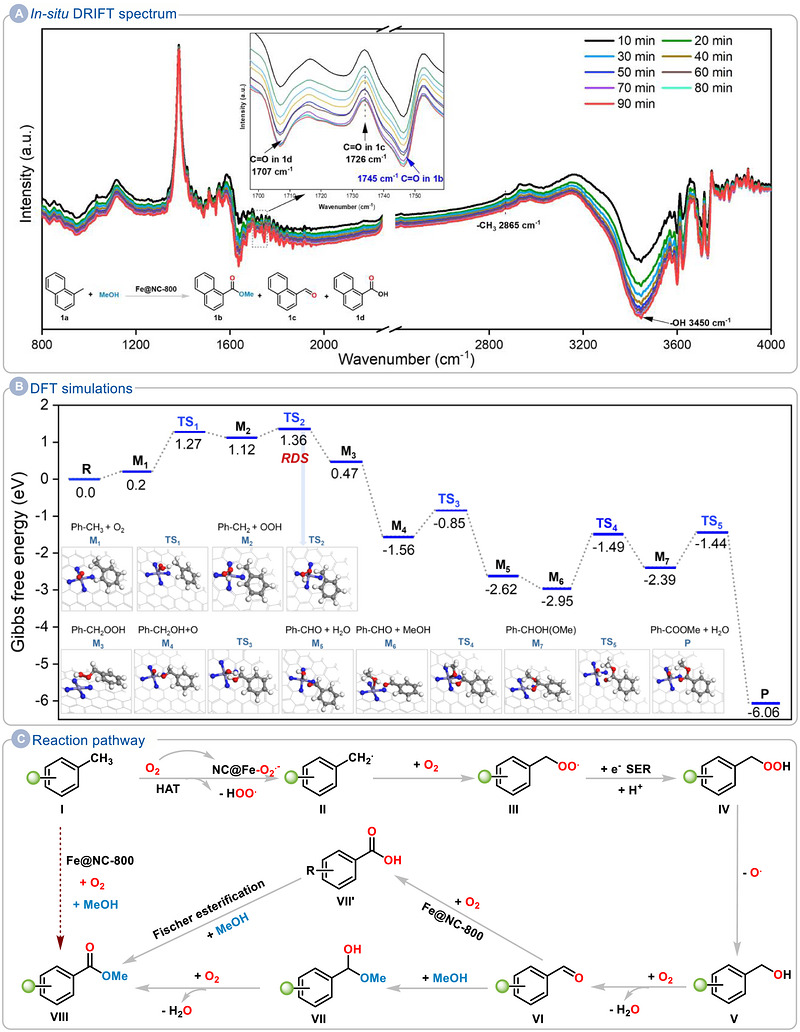
Spectroscopy Studies, DFT calculations and possible reaction pathway. (A) In situ DRIFT profile; (B) DFT simulations, purple (Fe), blue (N), red (O), gray (C), white (H); (C) Reaction pathway for Fe@NC‐800 catalyzed oxidative esterification of methyl arenes.

To gain deeper insight into the migration of intermediates and the reaction pathways, in situ diffuse reflectance infrared Fourier transform spectroscopy (DRIFTS) was performed under reaction conditions. Figure 8A shows the time‐resolved DRIFT spectra collected over a 90‐min period during the aerobic oxidation of **1a**. A distinct and progressive increase in the absorption band at approximately 1745 cm^−1^, attributed to the C═O stretching vibration of ester functionalities, indicates the gradual formation of methyl ester **1b** as the major product [44]. Simultaneously, a weaker but noticeable band emerges in the 1710–1725 cm^−1^ region, which can be assigned to the C═O stretches of aldehyde (**1c**) and/or carboxylic acid (**1d**) intermediates, reflecting their possible transient presence or limited accumulation throughout the reaction [46]. An increase in the 1250 cm^−1^ region, corresponding to C‐O stretching in esters, further corroborates the formation of esters. Overall, these spectral evolutions support a stepwise oxidation process wherein ester formation dominates, accompanied by minor or intermediate contributions from aldehyde and acid species.

Furthermore, to evaluate the thermodynamic feasibility and energy barriers of the key elementary steps involved in the oxidation process, density functional theory (DFT) calculations were performed using toluene as a model substrate. Detailed computational methods and parameters are provided in the Supporting Information (Section S5). As illustrated by the Gibbs free energy profile (Figure 8B), the transformation from toluene (**R**) to methyl benzoate (**P**) proceeds through a well‐defined multistep pathway. The reaction initiates with the co‐adsorption of O_2_ and toluene on the surface of the Fe@NC‐800 catalyst, exhibiting an adsorption energy of 0.2 eV (**M_1_**). Specifically, O_2_ is vertically adsorbed at the single Fe sites with an adsorption energy of −0.17 eV at 423 K, indicating a relatively weak yet favorable interaction (Figure S14). In this step, O_2_ undergoes single‐electron reduction (SER) at the Fe‐N site to form O_2_^●−^, which abstracts a H atom from toluene via transition state TS_1_, yielding a toluene radical and HOO• species, with a free energy change of +1.27 eV. These radical species (**M_2_**) then combine via TS_2_, identified as the rate‐determining step (RDS) with an energy barrier of 1.36 eV, to form a toluene hydroperoxide intermediate (**M_3_**, Ph‐CH_2_OOH), which then undergoes O─O bond cleavage to give benzyl alcohol (**M_4_**, Ph‐CH_2_OH, Δ*G* = −2.03 eV). Subsequent dehydrogenation of alcohol via TS_3_ affords the key intermediate benzaldehyde (**M_5_**, Ph‐CHO, −2.62 eV), overcoming an energy barrier of 1.06 eV, accompanied by the release of H_2_O. In the final stage, adsorbed MeOH undergoes O─H bond cleavage and couples with benzaldehyde (**M_6_**) through TS_4_ to form a hemiacetal intermediate (**M_7_**, Ph‐CH(OH)OMe), which ultimately dehydrogenates by TS_5_ to yield methyl benzoate (**P**, Ph‐COOMe). The overall reaction from toluene to methyl benzoate is highly exergonic, with a total free energy change of −6.06 eV, underscoring the thermodynamic favorability of the catalytic process.

Based on the results from kinetic and control experiments along with in situ experiments and DFT simulations, we propose the general reaction pathway for this Fe‐catalyzed CH‐oxidative esterification reaction. Initially, molecular oxygen is activated at the Fe‐N sites to generate a surface‐bound superoxide species (Fe‐O_2_•^−^), which abstracts a H atom from methyl arene (**I**) through a hydrogen atom transfer (HAT) process, affording a benzyl radical (**II**) and hydroperoxyl radical (HO_2_•). Then, benzyl radical rapidly couples with O_2_ to yield a benzyl peroxyl radical (**III**), which undergoes SER at the Fe@NC‐800 surface to form a benzyl peroxide anion. Subsequent protonation leads to benzyl hydroperoxide (**IV**), which undergoes homolytic O─O bond cleavage to produce benzylic alcohol (**V**). Benzylic alcohol is further oxidized by activated O_2_ to generate benzylic aldehyde (**VI**), which undergoes further oxidation to produce the corresponding acid (**VII’**); subsequently, the desired ester was achieved via the Fischer esterification process. On the other hand, the obtained aldehyde condensation with MeOH to form a hemiacetal intermediate (**VII**), followed by further oxidation to form the target ester (**VIII**, Figure 8C). In the case of esterification of alkylarenes via C─C bond cleavage, the RDS is the formation of the respective benzylic radicals, which leads to the formation of benzylic ketones (Figure 7A–5,A–6). The generated ketone then undergoes oxidative cleavage followed by esterification with MeOH to form the desired ester (Figure S15) [47, 48, 49].

## 3 Conclusions

In summary, we have developed an environmentally benign and sustainable protocol for the synthesis of (hetero)aromatic esters via a catalytic C─H oxidation strategy. This challenging transformation was achieved using an atomically dispersed iron catalyst featuring Fe‐N_4_ active sites anchored on N‐doped porous carbon. This Fe‐SAC system effectively overcomes the intrinsic inertness of benzylic C(sp^3^)─H and C─C bonds, enabling the oxidative esterification of a wide range of methyl (hetero)arenes, biomass‐derived platform chemicals, and even chemically resistant polystyrene plastic waste. In general, this methodology offers a green alternative to traditional esterification reactions by utilizing molecular oxygen as the oxidant and water as a solvent, thereby avoiding noble metals, harsh conditions, or toxic additives. Mechanistic investigations, supported by DFT simulations and in situ spectroscopy, reveal that the synergy between the isolated Fe sites and the N‐doped framework is pivotal for catalytic performance. Furthermore, this Fe‐SAC exhibited excellent stability and recyclability, highlighting its potential for industrial applications. This work not only demonstrates the power of single‐atom catalysis for oxidative functionalization but also provides a viable pathway for the upcycling of hydrocarbon feedstocks and polymeric materials into value‐added chemicals.

## Author Contributions

M.B., R.V.J., and Z.M. supervised the project. Z. M, Y.C., and B.Z. planned and developed the project and designed the experiments. Z.M. prepared catalysts and performed all catalytic experiments. Y.C. and B.Z. carried out the catalyst characterization and analysis. H.Y. performed DFT simulations. R.V.J., M.B., and Z.M. wrote the paper with the input and contribution of all authors.

## Conflicts of Interest

The authors declare no conflicts of interest.

## Supporting information




**Supporting File**: anie72942‐sup‐0001‐SuppMat.docx.

## Data Availability

The data that support the findings of this study are available from the corresponding author upon reasonable request.

## References

[1] R. Vanjari and K. N. Singh , “Utilization of Methylarenes as Versatile Building Blocks in Organic Synthesis,” Chemical Society Reviews 44 (2015): 8062–8096, 10.1039/C5CS00003C. 26377280

[2] L. Guillemard , N. Kaplaneris , L. Ackermann , and M. J. Johansson , “Late‐Stage C–H Functionalization Offers New Opportunities in Drug Discovery,” Nature Reviews Chemistry 5 (2021): 522–545, 10.1038/s41570-021-00300-6. 37117588

[3] K. Godula and D. Sames , “C–H Bond Functionalization in Complex Organic Synthesis,” Science 312 (2006): 67–72, 10.1126/science.1114731. 16601184

[4] T. Dalton , T. Faber , and F. Glorius , “C–H Activation: Toward Sustainability and Applications,” ACS Central Science 7 (2021): 245–261, 10.1021/acscentsci.0c01413. 33655064 PMC7908034

[5] L. Kesavan , R. Tiruvalam , M. H. A. Rahim , et al., “Solvent‐Free Oxidation of Primary Carbon‐Hydrogen Bonds in Toluene Using Au‐Pd Alloy Nanoparticles,” Science 331 (2011): 195–199, 10.1126/science.1198458. 21233383

[6] Z. Ma , N. Rockstroh , Z. Chen , et al., “Iron‐Based Single‐atom Catalysts for Selective Ammoxidation of C(*sp^3^ *)–H Bonds and Oxidative C–C Cleavage Reactions,” Nature Catalysis 9 (2026): 389–403, 10.1038/s41929-026-01513-y.

[7] E. Gaster , S. Kozuch , and D. Pappo , “Selective Aerobic Oxidation of Methylarenes to Benzaldehydes Catalyzed by N‐Hydroxyphthalimide and Cobalt(II) Acetate in Hexafluoropropan‐2‐ol,” Angewandte Chemie International Edition 56 (2017): 5912–5915, 10.1002/anie.201702511. 28436132

[8] J. P. Lumb , “Stopping Aerobic Oxidation in Its Tracks: Chemoselective Synthesis of Benzaldehydes From Methylarenes,” Angewandte Chemie International Edition 56 (2017): 9276–9277, 10.1002/anie.201704160. 28605566

[9] L. Wang , G. Wang , J. Zhang , C. Bian , X. Meng , and F.‐S. Xiao , “Controllable Cyanation of Carbon‐Hydrogen Bonds by Zeolite Crystals Over Manganese Oxide Catalyst,” Nature Communications 8 (2017): 15240, 10.1038/ncomms15240. PMC5440663 28504259

[10] D. Khalili , M. Rousta , A. Khalafi‐Nezhad , and E. Ebrahimi , “From Methylarenes to Esters: Efficient Oxidative Csp^3^–H Activation Promoted by CuO Decorated Magnetic Reduced Graphene Oxide,” New Journal of Chemistry 46 (2022): 14052–14064, 10.1039/D2NJ00728B.

[11] H. Su , K.‐X. Zhang , B. Zhang , et al., “Activating Cobalt Nanoparticles via the Mott–Schottky Effect in Nitrogen‐Rich Carbon Shells for Base‐free Aerobic Oxidation of Alcohols to Esters,” Journal of the American Chemical Society 139 (2017): 811–818, 10.1021/jacs.6b10710. 28006898

[12] D. S. Mannel , M. S. Ahmed , T. W. Root , and S. S. Stahl , “Discovery of Multicomponent Heterogeneous Catalysts via Admixture Screening: PdBiTe Catalysts for Aerobic Oxidative Esterification of Primary Alcohols,” Journal of the American Chemical Society 139 (2017): 1690–1698, 10.1021/jacs.6b12722. 28060501

[13] J. J. Li , “Fischer–Speier Esterification,” in Name Reactions: A Collection of Detailed Mechanisms and Synthetic Applications, 5th ed. (Springer, 2014), 252–252, 10.1007/978-3-319-03979-4.

[14] A. Mannu and A. Mele , “C–H Activation: Toward Sustainability and Applications,” Catalysts 14 (2024): 931, 10.3390/catal14120931.

[15] S. N. Khot , J. J. Lascala , E. Can , et al., “Development and Application of Triglyceride‐based Polymers and Composites,” Journal of Applied Polymer Science 82 (2001): 703–723, 10.1002/app.1897.

[16] R. Sang , P. Kucmierczyk , K. Dong , et al., “Palladium‐catalyzed Selective Generation of CO From Formic Acid for Carbonylation of Alkenes,” Journal of the American Chemical Society 140 (2018): 5217–5223, 10.1021/jacs.8b01123. 29528637

[17] J. Yang , J. Liu , H. Neumann , et al., “Direct Synthesis of Adipic Acid Esters via Palladium‐Catalyzed Carbonylation of 1,3‐Dienes,” Science 266 (2019): 1514–1517, 10.1126/science.aaz1293. 31857484

[18] E. Drent and P. H. Budzelaar , “Palladium‐Catalyzed Alternating Copolymerization of Alkenes and Carbon Monoxide,” Chemical Review 96 (1996): 663–682, 10.1021/cr940282j. 11848769

[19] J. Vondran , M. R. L. Furst , G. R. Eastham , et al., “Magic of Alpha: The Chemistry of a Remarkable Bidentate Phosphine, 1,2‐Bis(Di‐Tert‐Butylphosphinomethyl)Benzene,” Chemical Review 121 (2021): 6610–6653, 10.1021/acs.chemrev.0c01254. 33961414

[20] M. Liu , Z. Zhang , H. Liu , Z. Xie , Q. Mei , and B. Han , “Transformation of Alcohols to Esters Promoted by Hydrogen Bonds Using Oxygen as the Oxidant Under Metal‐Free Conditions,” Science Advances 4 (2018): eaas9319, 10.1126/sciadv.aas9319. 30310866 PMC6173529

[21] M. Liu , B. Han , and P. J. Dyson , “Simultaneous Generation of Methyl Esters and CO in Lignin Transformation,” Angewandte Chemie International Edition 61 (2022): e202209093, 10.1002/anie.202209093. 35979750 PMC9826404

[22] K. Suzuki , T. Yamaguchi , K. Matsushita , et al., “Aerobic Oxidative Esterification of Aldehydes With Alcohols by Gold–Nickel Oxide Nanoparticle Catalysts With a Core–Shell Structure,” ACS Catalysis 3 (2013): 1845–1849, 10.1021/cs4004084.

[23] S. Yamamatsu , T. Yamaguchi , K. Yokota , et al., “Development of Catalyst Technology for Producing Methyl Methacrylate (MMA) by Direct Methyl Esterification,” Catalysis Surveys from Asia 14 (2010): 124–131, 10.1007/s10563-010-9101-9.

[24] J. H. Teles , I. Hermans , G. Franz , and R. A. Sheldon , Oxidation, in Ullmann's Encyclopedia of Industrial Chemistry (Wiley‐VCH, 2015), 10.1002/14356007.a18_261.pub2.

[25] Z. Ma , C. Kuloor , C. Kreyenschulte , et al., “Development of Iron‐Based Single Atom Materials for General and Efficient Synthesis of Amines,” Angewandte Chemie International Edition 63 (2024): 8062–8096, 10.1002/anie.202407859. 38923207

[26] Z. Ma , B. Zhang , Z. He , et al., “Atomically Dispersed Fe‐N‐C‐Catalyzed Intermolecular Reductive Coupling Toward the Synthesis of Benzimidazoles,” ACS Catalysis 15 (2025): 11875–11885, 10.1021/acscatal.5c03077.

[27] H. Qi , S. Mao , J. Rabeah , et al., “Water‐Promoted Carbon‐Carbon Bond Cleavage Employing a Reusable Fe Single‐Atom Catalyst,” Angewandte Chemie International Edition 135 (2023): e202311913, 10.1002/ange.202311913. 37681485

[28] W. Liu , L. Zhang , X. Liu , et al., “Discriminating Catalytically Active FeN_x_ Species of Atomically Dispersed Fe‐N‐C Catalyst for Selective Oxidation of the C–H Bond,” Journal of the American Chemical Society 139 (2017): 10790–10798, 10.1021/jacs.7b05130. 28745500

[29] A. Wang , J. Li , and T. Zhang , “Heterogeneous Single‐Atom Catalysis,” Nature Reviews Chemistry 2 (2018): 65–81, 10.1038/s41570-018-0010-1.

[30] B. Singh , M. B. Gawande , A. D. Kute , et al., “Single‐Atom (Iron‐Based) Catalysts: Synthesis and Applications,” Chemical Reviews 121 (2021): 13620–13697, 10.1021/acs.chemrev.1c00158. 34644065

[31] Z. Li , S. Ji , Y. Liu , et al., “Well‐Defined Materials for Heterogeneous Catalysis: From Nanoparticles to Isolated Single‐Atom Sites,” Chemical Reviews 120 (2019): 623–682, 10.1021/acs.chemrev.9b00311. 31868347

[32] L. Liu and A. Corma , “Metal Catalysts for Heterogeneous Catalysis: From Single Atoms to Nanoclusters and Nanoparticles,” Chemical Reviews 118 (2018): 4981–5079, 10.1021/acs.chemrev.7b00776. 29658707 PMC6061779

[33] Z. Ma , Z. Chen , Z. Yuan , et al., “Synthesis of Aromatic Amides From Lignin and Its Derivatives,” Nature Communications 16 (2025): 3476, 10.1038/s41467-025-58559-y. PMC11992226 40216764

[34] K. Sun , H. Shan , H. Neumann , et al., “Efficient Iron Single‐atom Catalysts for Selective Ammoxidation of Alcohols to Nitriles,” Nature Communications 13 (2022): 1848, 10.1038/s41467-022-29074-1. PMC8986860 35387970

[35] Y. Jiang , S. Chen , Y. Chen , A. Gu , and C. Tang , “Sustainable Aerobic Allylic C–H Bond Oxidation With Heterogeneous Iron Catalyst,” Journal of the American Chemical Society 146 (2024): 2769–2778, 10.1021/jacs.3c12688. 38240486

[36] R. E. P. Chandrasena , K. P. Vatsis , M. J. Coon , et al., “Hydroxylation by the Hydroperoxy‐Iron Species in Cytochrome P450 Enzymes,” Journal of the American Chemical Society 126 (2004): 115–126, 10.1021/ja038237t. 14709076

[37] J. Qin , B. Han , X. Liu , W. Dai , et al., “An Enzyme‐Mimic Single Fe‐N_3_ Atom Catalyst for the Oxidative Synthesis of Nitriles via C–C Bond Cleavage Strategy,” Science Advances 8 (2022): eadd1267, 10.1126/sciadv.add1267. 36206338 PMC9544340

[38] M. Che and A. Tench , “Characterization and Reactivity of Molecular Oxygen Species on Oxide Surfaces,” Advances in Catalysis 32 (1983): 1–148.

[39] M. Anpo , G. Costentin , E. Giamello , H. Lauron‐Pernot , and Z. Sojka , “Characterisation and Reactivity of Oxygen Species at the Surface of Metal Oxides,” Journal of Catalysis 393 (2021): 259–280, 10.1016/j.jcat.2020.10.011.

[40] F. Jaouen , J. Herranz , M. Lefevre , J.‐P. Dodelet , et al., “Cross‐Laboratory Experimental Study of Non‐Noble‐Metal Electrocatalysts for the Oxygen Reduction Reaction,” ACS Applied Materials & Interfaces 1 (2009): 1623–1639, 10.1021/am900219g. 20355776

[41] W. Qiu , N. Yang , D. Luo , J. Wang , et al., “Precise Synthesis of Fe‐N_2_ With N Vacancies Coordination for Boosting Electrochemical Artificial N_2_ Fixation,” Applied Catalysis B: Environmental 293 (2021): 120216, 10.1016/j.apcatb.2021.120216.

[42] E. E. Kwon and J. Lee , “Polyethylene Terephthalate Production From a Carbon Neutral Resource,” Journal of Cleaner Production 469 (2024): 143210, 10.1016/j.jclepro.2024.143210.

[43] Z. Sun , B. Fridrich , A. de Santi , et al., “Bright Side of Lignin Depolymerization: Toward New Platform Chemicals,” Chemical Reviews 118 (2018): 614–678, 10.1021/acs.chemrev.7b00588. 29337543 PMC5785760

[44] K. P. Sullivan , A. Z. Werner , K. J. Ramirez , et al., “Mixed Plastics Waste Valorization Through Tandem Chemical Oxidation and Biological Funneling,” Science 378 (2022): 207–211, 10.1126/science.abo4626. 36227984

[45] B. Zhao , Z. Hu , Y. Sun , R. Hajiayi , T. Wang , and N. Jiao , “Selective Upcycling of Polyolefins Into High‐value Nitrogenated Chemicals,” Journal of the American Chemical Society 146 (2024): 28605–28611, 10.1021/jacs.4c07965. 39241040

[46] F. Herold , N. Oefner , D. Zakgeym , A. Drochner , W. Qi , and B. J. Etzold , “The High‐Temperature Acidity Paradox of Oxidized Carbon: An In Situ DRIFTS Study,” ChemCatChem 14 (2022): e202101586, 10.1002/cctc.202101586.

[47] X. Huang , X. Li , M. Zou , et al., “From Ketones to Esters by a Cu‐Catalyzed Highly Selective C(CO)–C(Alkyl) Bond Cleavage: Aerobic Oxidation and Oxygenation With Air,” Journal of the American Chemical Society 136 (2014): 14858–14865, 10.1021/ja5073004. 25251943

[48] H. Luo , L. Wang , S. Shang , et al., “Cobalt Nanoparticles‐Catalyzed Widely Applicable Successive C−C Bond Cleavage in Alcohols to Access Esters,” Angewandte Chemie International Edition 132 (2020): 19430–19436, 10.1002/ange.202008261. 32662588

[49] C. Xie , L. Lin , L. Huang , et al., “Zn‐N_x_ Sites on N‐Doped Carbon for Aerobic Oxidative Cleavage and Esterification of C(CO)–C Bonds,” Nature Communications 12 (2021): 4823, 10.1038/s41467-021-25118-0. PMC8355145 34376654

