# Supplementary material for: Single‐Atom Iron‐Catalyzed Oxidative Esterification: From Methylarenes to the Upcycling of Polystyrene and Lignin‐Derived Feedstocks
Source: Angew Chem Int Ed Engl. 2026 May 30;65(31):e6974259. doi: 10.1002/anie.6974259 (PMC13411391; doi:10.1002/anie.6974259)
Supplement: Supplementary file 1 — Supporting File: anie72942‐sup‐0001‐SuppMat.docx. [file ANIE-65-e6974259-s001.docx]

**Supporting information**

**Single-Atom Iron-Catalyzed Oxidative Esterification: From Methylarenes to the Upcycling of Polystyrene and Lignin-Derived Feedstocks**

Zhuang Ma,^1*+^ Binyu Zhang,^1,2+^ Hui Yang,^3+^ Yue Hu,^1^ Junzheng Yu,^2^ Yanbin Cui,^2^ Jun Zhao^4^, Matthias Beller,^1*^ Rajenahally V. Jagadeesh^1,4,5*^

^1^Leibniz-Institut für Katalyse e.V., Albert-Einstein-Str. 29a, Rostock, D-18059, Germany

^2^Guangzhou Institute of Energy Conversion, Chinese Academy of Sciences, Guangzhou, Guangdong 510640, China

^3^National Institute of Clean and Low Carbon Energy, Beijing, 102211, China

^4^Department of Biology, Hong Kong Baptist University, Hong Kong, SAR, China

^5^Nanotechnology Centre, Centre for Energy and Environmental Technologies (CEET), VŠB-Technical University of Ostrava, Ostrava-Poruba, Czech Republic

*Corresponding authors E-mails:

[ZhuangMaChem@hotmail.com](mailto:ZhuangMaChem@hotmail.com); [mathias.beller@catalysis.de](mailto:mathias.beller@catalysis.de); [jagadeesh.rajenahally@catalysis.de](mailto:jagadeesh.rajenahally@catalysis.de).

**Table of contents**

S1. Materials and methods

S2. Procedure for the preparation of catalysts

S3. General procedures for catalytic CH-oxidative esterification reactions

S4. Characterization of catalysts

S5. DFT calculations

S6. Possible reaction pathway for oxidative esterification of alkyl arenes

S7. NMR data

S8. NMR spectra

S9. References

**S1. Materials and methods**

All the substrates including methylarenes, alkylarenes and other chemicals were obtained commercially from different companies such as Sigma Aldrich, TCI chemicals, Alfa Aesar and BLDpharm. Iron (II) chloride tetrahydrate (cat no. 44939-250G), copper (II) chloride dihydrate (cat no. 459097-5G), nickel (II) chloride hexahydrate (cat no. 223387-25G), manganese (II) chloride tetrahydrate (cat no. 221279-100G), cobalt (II) chloride hexahydrate (cat no. 202185-100G), silica suspension (Silica LUDOX® HS-40 colloidal silica, cat no. 420816-1L, particle size ~12 nm) and 1,10-phenanthroline (cat no. P2099-25G) as well as 10wt% Pd/C and iron phthalocyanine (FePc; cat no. 379549-1G) were purchased from Sigma Aldrich. AEROSIL® 300 fumed silica (particle size ~7 nm) was acquired from Evonik. The pyrolysis experiments were carried out in Dekema Austromat 624 oven. Unless otherwise stated, all reagents were used directly without purification. All catalytic reactions were carried out in 300 mL or 25 mL autoclave (PARR Instrument Company). To avoid unspecific reactions, all catalytic reactions were carried out either in glass vials, which were placed inside the autoclave, or glass vessel fitted autoclaves.

XRD powder pattern were recorded on a Panalytical X'Pert diffractometer equipped with a Xcelerator detector using automatic divergence slits and Cu kα1/α2 radiation (40 kV, 40 mA; λ= 0.15406 nm, 0.154443 nm). Cu beta-radiation was excluded using a nickel filter foil. The measurements were performed in 0.0167° steps and 100 s of data collecting time per step. The samples were mounted on silicon zero background holders. The obtained intensities were converted from automatic to fixed divergence slits (0.25°) for further analysis. Peak positions and profile were fitted with Pseudo-Voigt function using the High Score Plus software package (Panalytical). Phase identification was done by using the PDF-2 database of the International Center of Diffraction Data (ICDD).

Scanning Electron Microscope (SEM) and field Emission Transmission Electron Microscope (FETEM) images were acquired on SU-70 (Hitachi) and JEM-2100F (JEOL) instruments, respectively. AC-TEM images were taken by JEM-ARM200F Atomic Resolution Analytical Electron Microscope (JEOL).

Raman spectra were recorded on WITec alpha300R with a 532 nm laser.

Surface area and pore size measurements were performed with N_2_ adsorption isotherms by Micromeritics ASAP 2460 at 77 K. The specific surface area was confirmed by the Brunauer-Emmett-Teller (BET) equation.

XPS surface investigation has been performed on the PHI 5000 Versa Probe II XPS system (Physical Electronics) with monochromatic Al-Kα source (15 kV, 50 W) and photon energy of 1486.7 eV. Dual beam charge compensation was used for all measurements. All the spectra were measured in the vacuum of 1.3 x 10^-7^ Pa and at the room temperature of 21 °C. The analyzed area on each sample was spot of 200 µm in diameter. The survey spectra were measured with pass energy of 187.850 eV and electron volt step of 0.8 eV while for the high-resolution spectra was used pass energy of 23.500 eV and electron volt step of 0.2 eV. The spectra were evaluated with the MultiPak (Ulvac - PHI, Inc.) software. All binding energy (BE) values were referenced to the carbon peak C 1s at 284.80 eV.

The transmission ^57^Fe Mössbauer spectrum was collected employing a Mössbauer spectrometer operating at a constant acceleration mode and equipped with 50 mCi ^57^Co(Rh) source. For fitting the Mössbauer spectrum, the MossWinn software program was used.^1^ The isomer shift values are referred to α-Fe at room temperature.

EPR spectra were collected on X-band (∼9.14-9.17 GHz) spectrometer JEOL JES-X-320 equipped with variable He temperature set-up ES-CT470 apparatus. A high-purity quartz tube (Suprasil, Wilmad, ≤0.5 OD) was used as a sample holder; the accuracy of the *g*-values was determined by comparison with a Mn^2+/^MgO standard (JEOL standard). The microwave power was set to 1.0 mW to avoid any power saturation effects. A modulation width of 1 mT and a modulation frequency of 100 kHz was used. All EPR spectra were collected with a time constant of 30 ms and a sweep time of 4 min with 5 accumulations to improve the signal-to-noise ratio.

The X-ray absorption fine structure (XAFS) spectra were measured in fluorescence mode at beamline BL14W1 of the Shanghai Synchrotron Radiation Facility (SSRF), China. The storage rings were operated at 3.5 GeV with a 230-mA electron beam current. The beamline was monochromatized with a double-crystal monochromator (DCM) equipped with Si (111) crystals and the beam size at the samples was approximately 300 μm×300 μm (FWHM). Each spectrum was recorded over about 32 min with a focused beam. All XAFS spectra were analyzed using the Demeter software package (University of Chicago). The spectra were calibrated, averaged, pre-edge background subtracted, and post-edge normalized using the Athena program. The Fourier transformation of the *k*^3^-weighted EXAFS oscillations, *k*^3^·χ(k), from k space to R space was performed to obtain a radial distribution function. The data fitting was performed using the Artemis program.

Electron Paramagnetic Resonance (EPR) spectroscopy was performed on a Bruker EMX plus-6/1 at the X-band with a field modulation of 100 kHz. Briefly, 10 mg Fe@NC-800 with 5 mL methanol were mixed in a beaker, heating to 150 °C and filling with O_2_ for 10 minutes. After the reaction, the spin-trapping reagent DMPO was added to the reaction mixture which was measured in a glass capillary tube by EPR spectroscopy.

For the O_2_-temperature programmed desorption (O_2_-TPD) experiments, 50 mg of the iron sample was initially heated from room temperature to 200 °C at a rate of 10 °C/min under a helium (He) flow (50 mL/min) for 1 hour to ensure thorough drying and pretreatment. Following this, the sample was cooled to 50 °C, and a 10% O_2_/He gas mixture (50 mL/min) was introduced for 1 hour to achieve oxygen saturation on the sample surface. Subsequently, the system was purged with pure He (50 mL/min) for an additional hour to remove weakly physiosorbed oxygen species. Finally, the temperature was ramped to 800 °C at a rate of 10 °C/min under a continuous He flow, and the desorbed gases were monitored using a thermal conductivity detector (TCD).

GC and GC-MS analysis were recorded on Agilent 6890N instrument. GC conversion and yields were determined by GC-FID, HP6890 chromatograph with FID detector, column HP 530 m x 250 mm x 0.25 μm. NMR spectra are recorded using Bruker 300 Fourier, Bruker AV 300, and Bruker AV 400 spectrometers. Chemical shifts are reported in ppm relative to the deuterated solvent. Coupling constants are expressed in Hertz (Hz). The following abbreviations are used: s = singlet, bs = broad singlet d = doublet, t = triplet and m = multiple. The residual solvent signals were used as reference chemical shifts for ^1^H and ^13^C NMR spectra (CDCl_3_: δH = 7.26 ppm, δC = 77.12 ppm; DMSO-d_6_: δH = 2.50 ppm, δC = 39.52 ppm).

**S2. Procedure for the preparation of catalysts**

In a 100 mL dried round bottomed flask, FeCl_2_^.^4H_2_O (2.0 mmol; 398 mg) and 1,10-phenanthroline (**Phen**; 4.0 mmol; 721 mg) were dissolved in 30 mL EtOH by stirring for 1 h at 60 °C. Then, 3.5 g colloidal silica (Silica LUDOX® HS-40) was added and continued stirring further for 15 h at 60 °C. Subsequently, the solvent was removed by rotary evaporation and obtained solid material was dried in an oven overnight. Then, this dried material was transferred to crucible, and the crucible was closed with a lid and placed in a pyrosis oven and then heated to the defined temperature (400, 600, 800, 1000 °C) for 2 h at the heating rate of 5 °C/min under argon gas. After the completion of pyrolysis, the oven was cooled down to room temperature and the material was removed from the oven (Fe@NC-SiO_2_-800). Next, the obtained pyrolyzed materials were etched in 5 M NH_4_HF_2_ aqueous solution at room temperature for 24 h to remove the SiO_2_ template and any large particles presented. Finally, the resulting catalytic materials were filtered and washed subsequently with deionized water and then ethanol three times and finally dried under vacuum. These catalysts were represented as named Fe@NC-T, where T represents the pyrolysis temperature. Elemental analysis of optimal catalyst, Fe@NC-800: C = 64.1 wt.%, N = 8.5 wt.%, H = 0.72 wt.%, Si = 0.30 wt.%. Fe@NC-800-R: C = 65.3 wt.%, N = 8.2 wt.%, H = 0.7 wt.%, Si = 0.31 wt.%. The iron content was measured by ICP-OES to be approximately 1.32 wt% and 1.31 wt.% in the fresh (Fe@NC-800) and recycled (Fe@NC-800-R; after one run) catalysts, respectively.

Similar procedure has been applied for the preparation of other catalytic materials using different metal salts such as Mn@NC-800, Co@NC-800, Cu@NC-800, Ni@NC-800 and Pd@NC-800 using MnCl_2_·H_2_O, CoCl_2_·6H_2_O, CuCl_2_·2H_2_O, NiCl_2_·6H_2_O and PdCl_2_, respectively.

Fe@NC materials with different Fe loadings were prepared following the same procedure as Fe@NC-800, except that different amounts of FeCl_2_·4H_2_O were used. Specifically, 0.2, 1.0, and 4.0 mmol of FeCl_2_·4H_2_O were employed, and the resulting materials were denoted as Fe_0.2_@NC-800, Fe_1_@NC-800, and Fe_4_@NC-800, respectively. ICP-OES analysis showed that the Fe loadings of these samples were 0.38, 0.91, and 2.86 wt%, respectively.

Fe@NC-800-7nm was prepared using the same procedure as Fe@NC-800, except that AEROSIL® 300 fumed silica (particle size ~7 nm) was used instead of colloidal silica (LUDOX® HS-40) as the hard template. The Fe loading was measured by ICP-OES to be 1.35 wt%.

**S3.** **General procedures for catalytic CH-oxidative esterification reactions**

*S3.1 General procedure for the synthesis of aromatic esters from methyl (alkyl)arenes and MeOH*

A magnetic stirring bar, 0.2 mmol methylarene (or alkylarene) and 30 mg Fe@NC-800 catalyst (3.55 mol% Fe) were transferred to 8 mL glass vials. Next, 2.5 mL deionized H_2_O and 0.5 mL MeOH were added, and the vial was fitted with septum, cap, and needle. The reaction vials (8 vials with different substrates at a time) were placed into a 300 mL autoclave. The autoclave was flushed with nitrogen (with 10 bar) twice and O_2_ (5 bar) twice and then it was pressurized with 5 bar of O_2_. The autoclave was placed into an aluminum block preheated at 150-160 °C and the reactions were stirred for the required time. After completion of reactions, the autoclave was cooled to room temperature. The remaining O_2_ was discharged and the vials containing reaction products were removed from the autoclave. The solid catalyst was filtered off and washed thoroughly with ethyl acetate. The products in the filtrate were extracted with ethyl acetate and analyzed by GC and GC-MS. The corresponding esters were purified by column chromatography. For selected products, yields were determined by GC. For GC analysis, the filtrate containing products were extracted with ethyl acetate. To this ethyl acetate solution containing products, and mesitylene (0.2 mmol) as standard was added and then products were quantified by GC analysis. To ensure reproducibility, all the catalytic experiments were performed at least in duplicate.

TOF value calculation based on the following formula:

TOF (h^-1^) = $\frac{moles of product formed (mmol)}{moles of metal used \left( mmol \right)*reaction time (h)}$

**Table S1.** Oxidative esterification of 1-methylnaphthalene with methanol to methyl 1-naphthoate. Testing commercial catalysts.

| **Entry** | **Catalysts** | **Conv.1a (%)** | **Yield of 1b (%)** | **Yield of 1c (%)** | **Yield of 1d (%)** |
| --- | --- | --- | --- | --- | --- |
| 1 | Fe@SiO_2_-800 | <10 | <5 | <5 | <5 |
| 2 | FeCl_2_ + Phen | 10 | <5 | <5 | <5 |
| 3 | Rh/C | <10% | <2% | <5% | <5% |
| 4 | Pt/C | <10% | <2% | <5% | <5% |
| 5 | Pd/Al_2_O_3_ | <10% | <2% | <5% | <5% |
| 6 | Pd/C | 5 | - | - | - |
| 7 | Ru/C | 6 | 1 | 2 | 2 |
| 8 | FePC | 5 | - | - | - |
| 9 | Nano CuO | 17 | 5 | 2 | 3 |
| 10 | Fe_0.2_@NC-800 | 81 | 63 | 4 | 6 |
| 11 | Fe_1_@NC-800 | 92 | 71 | 5 | 9 |
| 12 | Fe@NC-800 | 95 | 77 | 7 | 8 |
| 13 | Fe_4_@NC-800 | 84 | 65 | 6 | 7 |
| 14 | Fe@NC-800-7nm | 73 | 56 | 4 | 5 |

**Reaction conditions:** 0.2 mmol 1-methylnaphthalene, 3.5 mol% metal basis, 5 bar O_2_, 2.5 mL H_2_O, 0.5 mL MeOH (~ 62 equiv.), 150 °C, 24 h. For homogeneous catalysis conditions: 10 mol% of FeCl_2_**·**4H_2_O and 30 mol% of **Phen** were used. Conversion and yield were obtained by GC using mesitylene as internal standard.

**Table S2.** Oxidative esterification of 1-methylnaphthalene with methanol to methyl 1-naphthoate. Testing of different solvents_._

| **Entry** | **Solvent** | **Conv.1a (%)** | **Yield of 1b (%)** | **Yield of 1c (%)** | **Yield of 1d (%)** |
| --- | --- | --- | --- | --- | --- |
| 1 | H_2_O | 95% | 77% | 7% | 8% |
| 2 | MeCN | 39% | 15% | 6% | 8% |
| 3 | t-BuOH | 29% | 10% | 11% | 5% |
| 4 | 1.4-dioxane | 16% | 7% | 4% | 3% |
| 5 | DMF | 19% | 6% | 5% | 4% |
| 6 | DMSO | <10% | <5% | <5% | <5% |
| 7 | MeOH | <15% | <5% | <5% | <5% |

**Reaction conditions:** 0.2 mmol 1-methylnaphthalene, 30 mg Fe@NC-800 (3.55 mol% Fe), 5 bar O_2_, 2.5 mL solvent, 0.5 mL MeOH (~ 62 equiv.), 150 °C, 24 h, Conversion and yield were obtained by GC using mesitylene as internal standard.

**Table S3.** Oxidative esterification of 1-methylnaphthalene with methanol to methyl 1-naphthoate. Testing of different amounts of MeOH.

| **Entry** | **MeOH amount** | **Conv.1a (%)** | **Yield of 1b (%)** | **Yield of 1c (%)** | **Yield of 1d (%)** |
| --- | --- | --- | --- | --- | --- |
| 1 | 0.1 mL | 93% | 19% | 40% | 31% |
| 2 | 0.3 mL | 90% | 43% | 24% | 20% |
| 3 | 0.5 mL | 95% | 77% | 7% | 8% |
| 4 | 1 mL | 57% | 26% | 14% | 15% |
| 5 | 2 mL | 31% | 8% | 15% | 7% |

**Reaction conditions:** 0.2 mmol 1-methylnaphthalene, 30 mg Fe@NC-800 (3.55 mol% Fe), 5 bar O_2_, 3 mL H_2_O + MeOH, 150 °C, 24 h, Conversion and yield were obtained by GC using mesitylene as internal standard.

**Table S4.** Oxidative esterification of 1-methylnaphthalene with methanol to methyl 1-naphthoate. Testing of different temperature and oxygen pressure.

| **Entry** | **Temp. (°C),**  **O_2_ (bar)** | **Conv.1a (%)** | **Yield of 1b (%)** | **Yield of 1c (%)** | **Yield of 1d (%)** |
| --- | --- | --- | --- | --- | --- |
| 1 | 150 °C, 5 bar | 95% | 77% | 7% | 8% |
| 2 | 130 °C, 5 bar | 60% | 38% | 10% | 10% |
| 3 | 150 °C, 1 bar | <10% | - | - | - |
| 4 | 150 °C,  20 bar air | 39% | 11% | 14% | 10% |

**Reaction conditions:** 0.2 mmol 1-methylnaphthalene, 30 mg Fe@NC-800 (3.55 mol% Fe), 2.5 mL H_2_O, 0.5 mL MeOH (~ 62 equiv.), 24 h, Conversion and yield were obtained by GC using mesitylene as internal standard.

**Table S5**. State-of-the-art for the oxidative esterification of methylarenes or alcohols with heterogeneous materials.

| **Entry** | **Catalyst** | **T (°C) / t (h)** | **O_2_ (bar)** | **TOF**  **(h^-1^)** | **Product** | **Yield (%)** | **Ref.** |
| --- | --- | --- | --- | --- | --- | --- | --- |
|  | | | | | | | |
| 1 | Fe@NC-800 | 150/24 | 5 bar O_2_ | 0.91 |  | 77% | This work |
|  |  |  |  | 0.95 |  | 81% |  |
| 2 | Au-Pd/C | 160/7 | 10 bar O_2_ | 83 |  | 49% | 2 |
| 3 | rGO/Fe_3_O_4_-CuO | 80/10 | TBHP, TBAB | <1 |  | 82% | 3 |
|  | | | | | | | |
| 4 | PdBi_0.47_Te_0.09_/C, 5 wt% Pd | 60/8 | 1 bar O_2_ | - |  | 99% | 4 |
| 5 | Co@NC | 60/12 | 1 bar O_2_ | 1.5 |  | 99% | 5 |
| 6 | Co_3_O_4_-N@C | 60/24 | 1 bar O_2_ | 1.58 |  | 95% | 6 |
| 7 | Co@C-N | 60/96 | 1 bar air | 0.07 |  | 99% | 7 |
| 8 | Ionic liquids | 90/24 | 2 MPa O_2_ | - |  | 70% | 8 |
| 9 | AuNiOx | 80/1 | 3 MPa 7% O_2_ | - |  | 59% | 9 |
| 10 | Au-NPs | 130/4 | 3 atm O_2_ | - |  | 100% | 10 |

**Table S6.** Oxidative esterification of 1-methylnaphthalene with methanol to methyl 1-naphthoate. Analysis of methanol oxidation products.

| **Entry** | **MeOH amount** | **Conv. 1a (%)** | **Yield of 1b (%)** | **Conv. MeOH (%)** | **Yield of MF (%)** | **Yield of HCHO (%)** | **Yield of FA (%)** |
| --- | --- | --- | --- | --- | --- | --- | --- |
| 1 | 1 mL | 89% | 70% | 21% | 3% | 5% | 8% |

**Reaction conditions:** 0.5 mmol 1-methylnaphthalene, 75 mg Fe-NC-800, 5 bar O_2_, 5 mL H_2_O, 1 mL MeOH (~ 50 equiv.), 150 °C, 24 h. Conversions and yields were determined by GC using mesitylene as internal standard. The yields of MF, HCHO, and FA were calculated based on the initial amount of MeOH. The reaction was performed in 25 mL parr autoclave with glass lined.

*S3.2 General procedure for the recycling experiment*

The magnetic stirring bar, 0.5 mmol 1-methylnaphthalene, 75 mg Fe@NC-800 (3.55 mol% Fe) were transferred to 25 mL parr autoclave fitted with glass vessel, then 5 mL deionized H_2_O and 1 mL MeOH were added. Subsequently, the autoclave was sealed and flushed with nitrogen (with 10 bar) twice and O_2_ (with 5 bar) twice and then it was pressurized with 5 bar of O_2_. The autoclave was placed into an aluminum block preheated at 150 °C and the reactions were stirred for the required time. After the completion of the reactions, the autoclave was cooled to room temperature. The remaining O_2_ was discharged, and the products were removed from the autoclave. The catalyst was separated by centrifugation, and the separated material was washed with deionized water, ethanol and ethyl acetate. Finally, the recycled catalyst was dried and used for the next run without further purification or reactivation. For the product analysis, the centrifugate containing products was extracted with ethyl acetate and then mesitylene as standard was added to the ether layer and analyzed finally by GC and GC-MS.

*
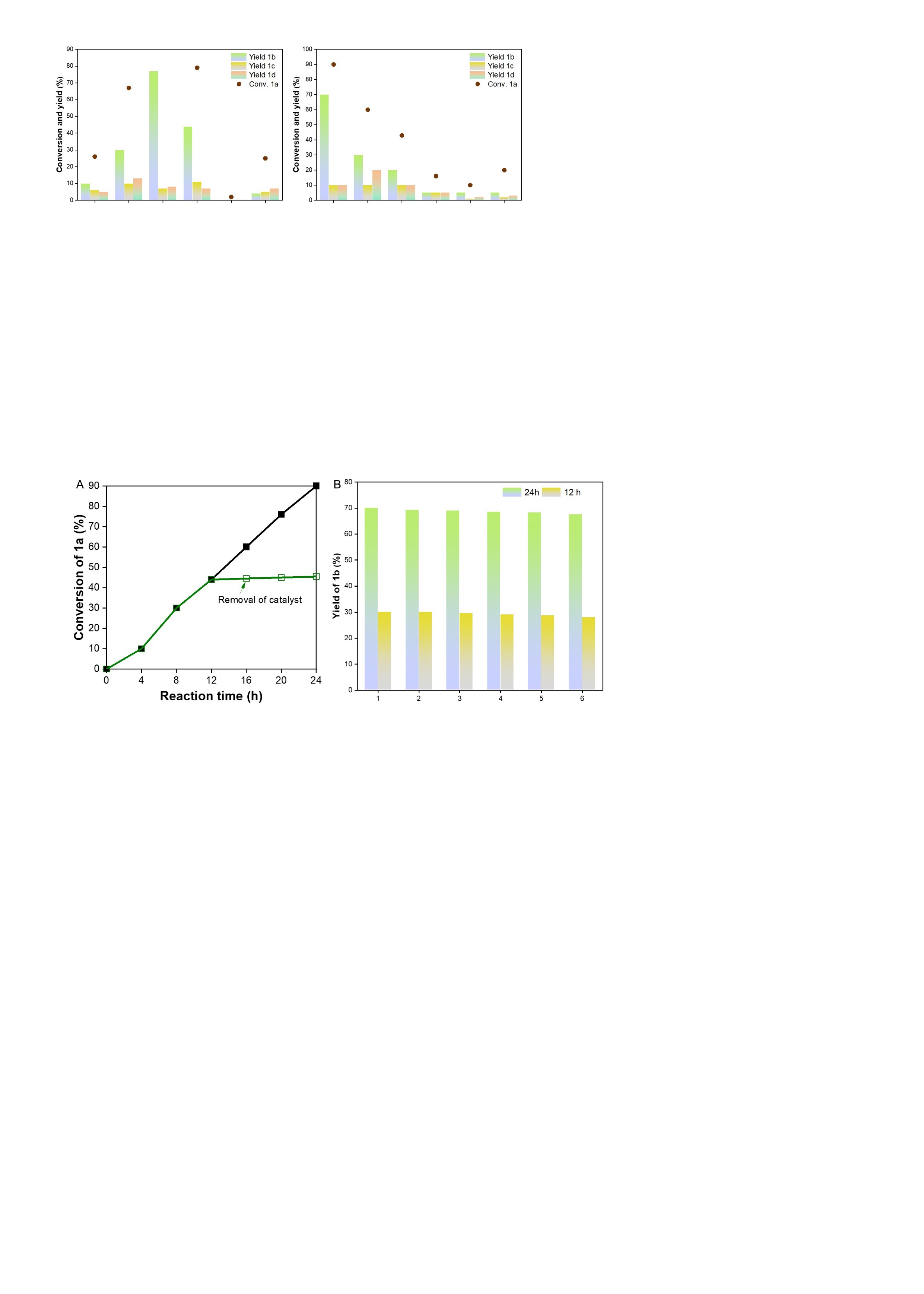
*

**Fig. S1. Hot filtration and recycling experiments.** Reaction conditions for hot filtration experiment: 0.2 mmol 1-methylnaphthalene, 30 mg Fe@NC-800 (3.55 mol% Fe), 5 bar O_2_, 2.5 mL H_2_O, 0.5 mL MeOH (~ 62 equiv.), 150 °C; Reaction conditions for recycle experiment: 0.5 mmol 1-methylnaphthalene, 75 mg Fe-NC-800, 5 bar O_2_, 5 mL H_2_O, 1 mL MeOH (~ 50 equiv.), 150 °C, 12 h or 24 h. Conversion and yields were determined by GC using mesitylene as internal standard.

*S3.3 General procedure for long-term stability test of Fe@NC-800 with flow reaction*

Evaluation of the long-term stability of Fe@NC-800 was carried out in a fixed bed reactor. 200 mg Fe@NC-800 and 300 mg quartz sand (40-60 mesh) were mixed and packed in a fixed bed reactor (internal diameter 5 mm，length 30 cm). Subsequently, 0.02 M lignin model compound (**21e**) and 0.45 M aqueous methanol in MeCN were pumped into the reactor (0.1 mL/min) at a weight hourly space velocity (WHSV) of 0.129 h^-1^ and 0.5 MPa O_2_ with 70 mL/min. The temperature of the fixed bed was set at 140°. Afterward, the products were separated by a gas-liquid separator, and the liquid products were collected. The conversion, yield and selectivity were determined by GC (Shimadzu GC-2014 equipped with FID detector), column SH-Rxi-5Sil with 30 m x 250 μm.

**S4. Characterization of catalysts**

*XRD patterns*

**Fig. S2.** XRD pattern of Fe@NC-800 and recycled one.

*Raman spectra*

**Fig. S3.** Raman spectra of Fe@NC-800.

*O_2_-TPD profile*

**Fig. S4.** O_2_-TPD profile of Fe@NC-800 at (A) different temperature and (B) different time.

*BET analysis*

**Fig. S5.** Nitrogen adsorption-desorption isotherms (A) and pore diameter (B) of Fe@NC-800 and Fe@NC-SiO_2_-800.

**Table S7.** BET surface area of Fe@NC-800 and recycled one.

| **Sample** | **S_BET_(m²/g)** | **V_t_(cm³/g)** | **D_ave_(nm)** |
| --- | --- | --- | --- |
| Fe@NC-800 | 827.4 | 0.735 | 10.6 |
| Fe@NC-SiO_2_-800 | 12.3 | 0.124 | 3.6 |

S_BET_: specific surface area measured by the BET method; V_t_: total pore volume; D_ave_: average pore diameter.

*STEM images*

**
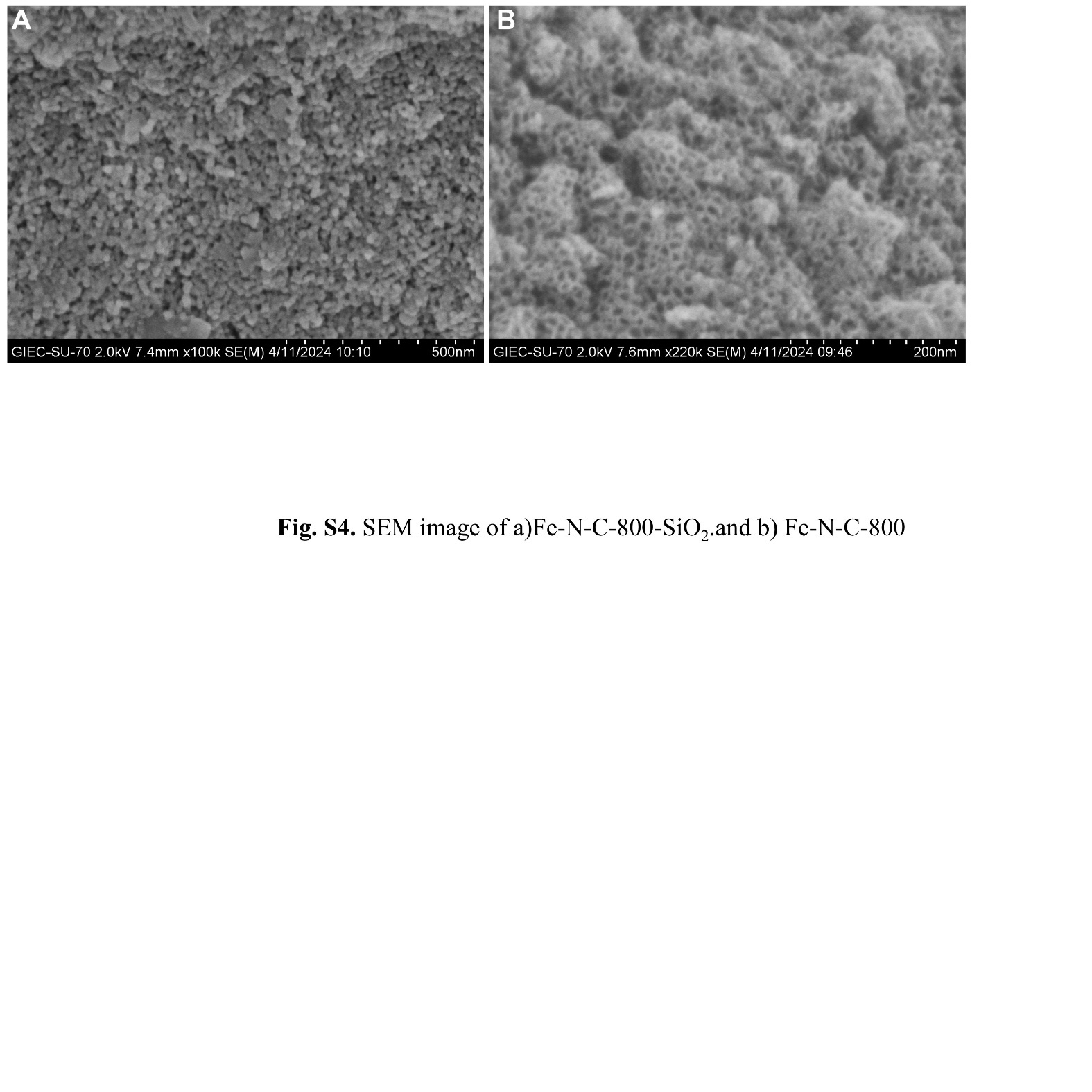
**

**Fig. S6.** SEM images of A) Fe@NC-800-SiO_2_ and B) Fe@NC-800.


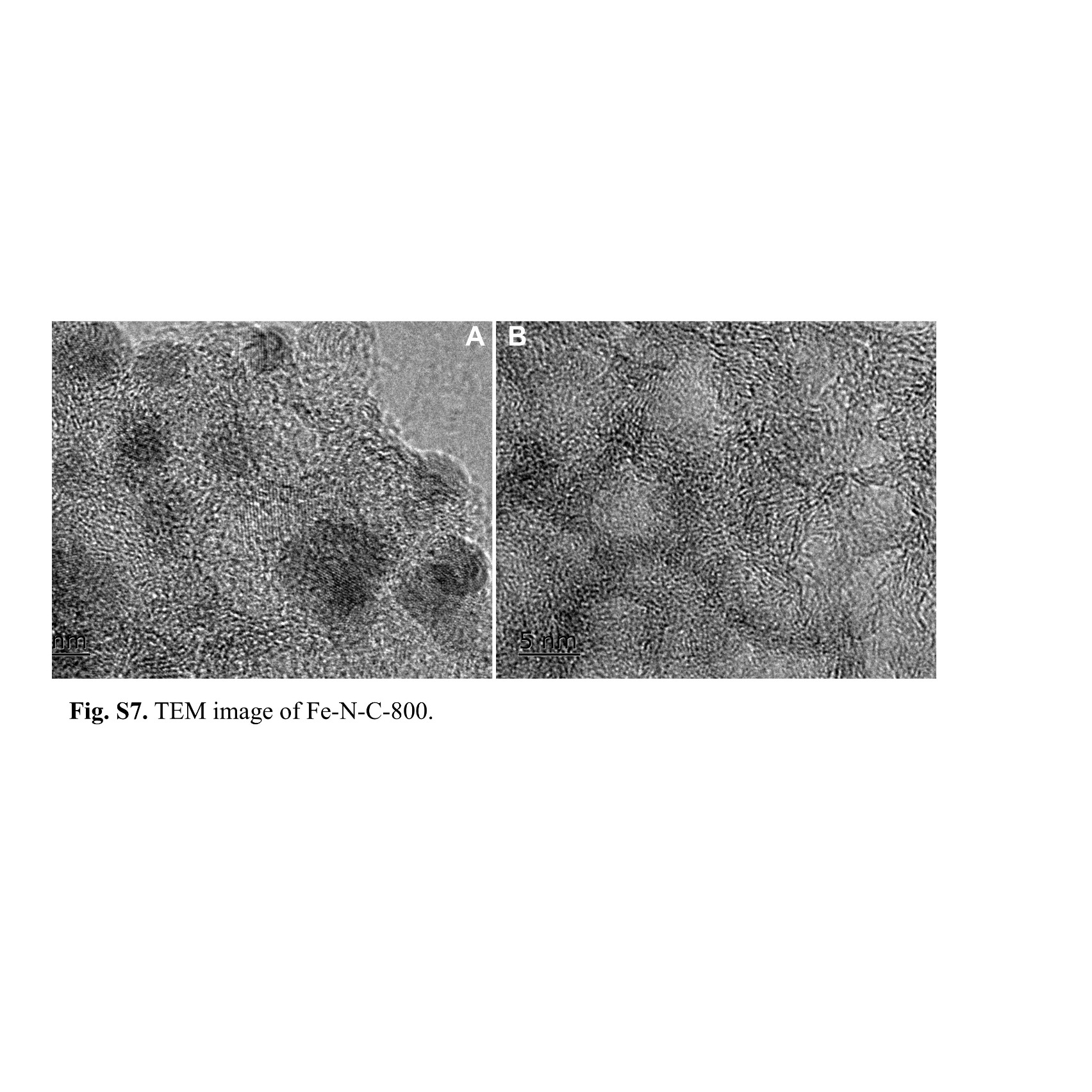


**Fig. S7.** TEM images of Fe@NC-800.


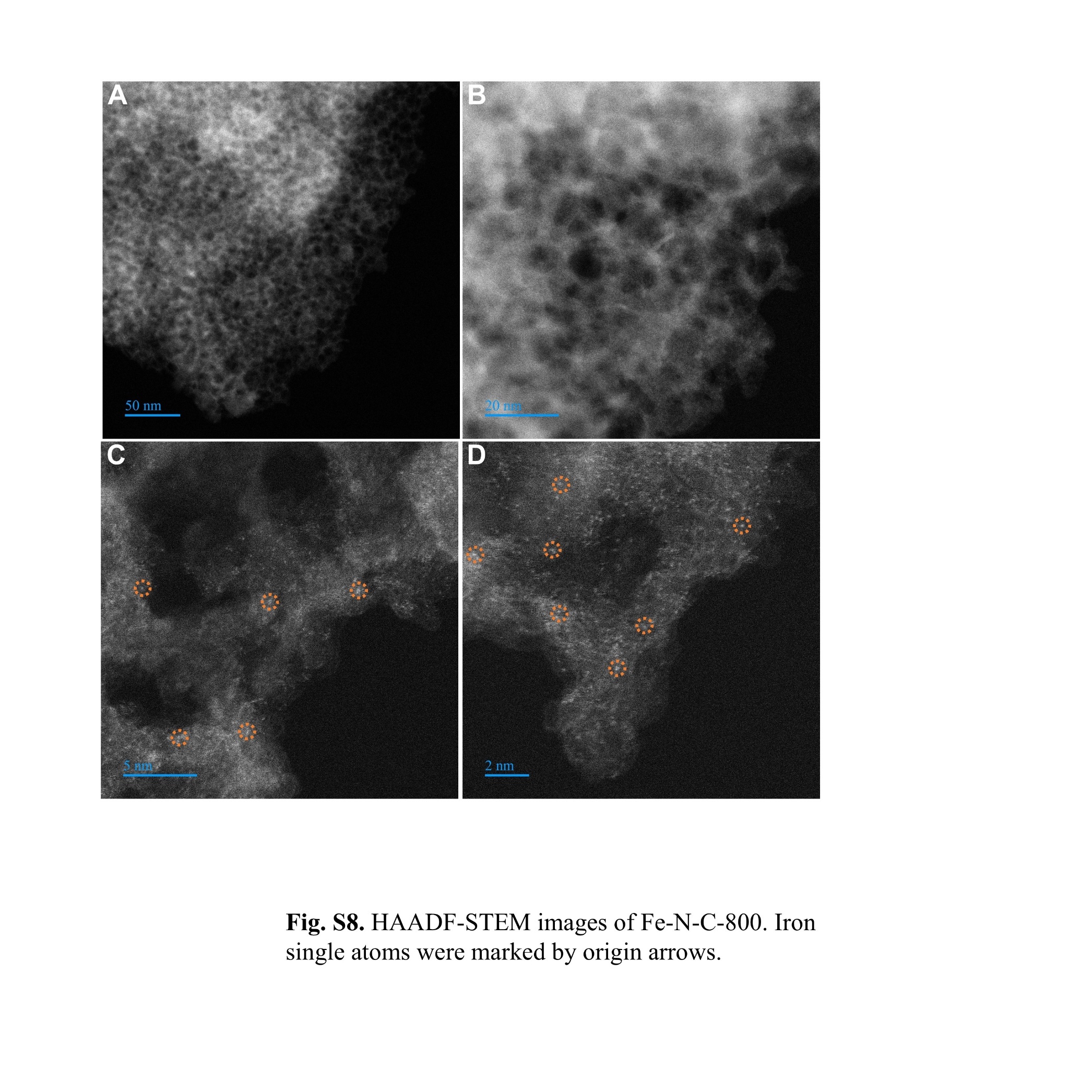


**Fig. S8.** HAADF-STEM images of Fe@NC-800. Iron single atoms were marked by circles.


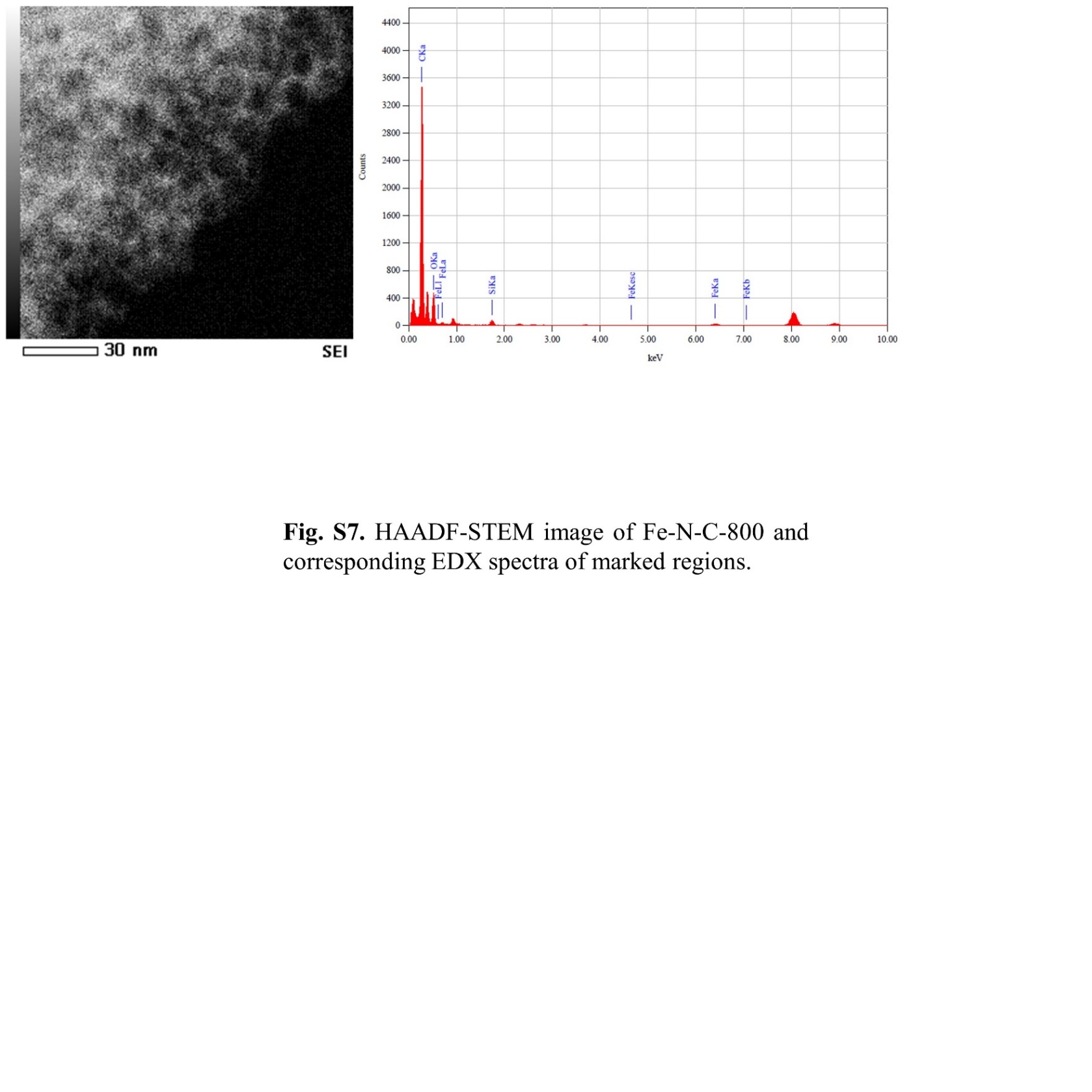


**Fig. S9.** HAADF-STEM image of Fe@NC-800 and corresponding EDX spectra.

*XPS spectra*

**Table S8.** Surface composition of different cobalt samples obtained by XPS. (All values are given in at.%.)

| **Catalyst** | **C** | **O** | **N** | **Fe** | **Si** | **F** |
| --- | --- | --- | --- | --- | --- | --- |
| Fe@NC-800 | 80.7 | 9.1 | 9.2 | 0.2 | 0.5 | 0.2 |
| Fe@NC-800-R | 80.9 | 8.7 | 8.7 | 0.2 | 0.8 | 0.2 |

**Fig. S10.** XPS survey spectrum of the Fe@NC-800 and recycled one.

**Fig. S11.** XPS of C 1s (A) and O 1s (B) region of Fe@NC-800.

**Fig. S12.** XPS of C 1s (A), O 1s (B), N 1s (C) and Fe 2p (D) region of Fe@NC-800-R (reused Fe@NC-800).

*XAS analysis*

***
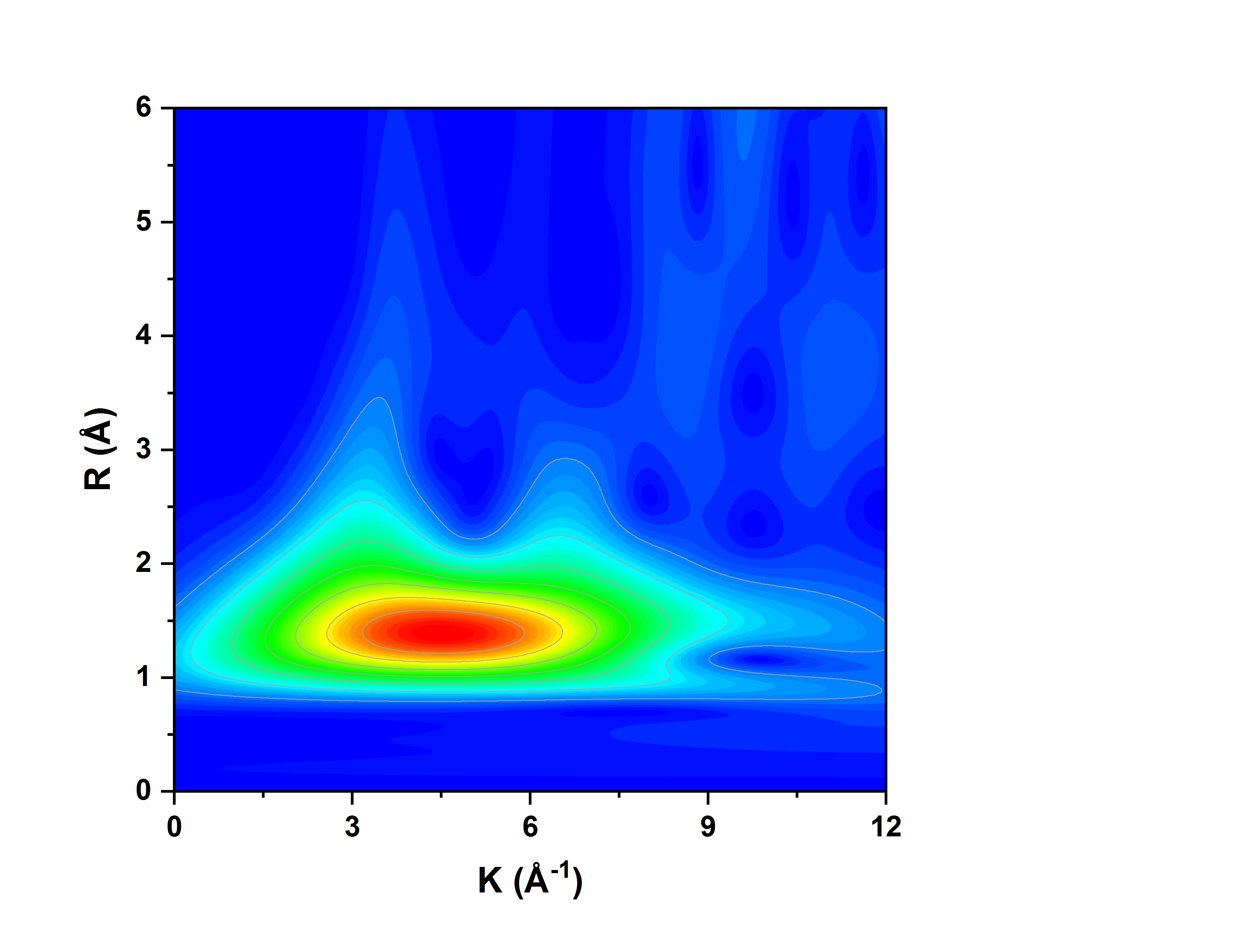
***

**Fig. S13.** Wavelet transform of Fe@NC-800.

**Table S9.** Fitting parameters for Fe K-edge EXAFS for the samples.

| **Sample** | **Shell** | **CN^a^** | **R(Å)^b^** | **σ^2^(Å^2^)^c^** | **ΔE_0_(eV)^d^** | **R factor** |
| --- | --- | --- | --- | --- | --- | --- |
| **Fe@NC-800** | Fe-N | 3.8 ± 0.2 | 2.01 | 0.012 | -0.061 | 0.029 |
| **Fe foil** | Fe-Fe | 5.68 | 2.46 | 0.006 | 3.594 | 0.005 |
|  | Fe-Fe | 4.26 | 2.84 | 0.005 | 3.273 | 0.005 |

*^a^CN*, coordination number; *^b^R*, the distance to the neighboring atom; *^c^σ*^2^, the Mean Square Relative Displacement (MSRD); *^d^ΔE*_0_, inner potential correction; *R* factor indicates the goodness of the fit. *S*0^2^ was fixed to 0.7726, according to the experimental EXAFS fit of the sample foil by fixing *CN* as the known crystallographic value. This value was fixed during EXAFS fitting, based on the known structure of Fe foil. Data range 3.0 ≤ k ≤ 11.0 Å^-1^, 1.0 ≤ R ≤ 2.0 Å. The Debye-Waller factors and Δ*R*s are based on the *guessing* parameters and constrained for paths.

**S5. DFT calculations**

All spin-polarized energy and frequency calculations were performed using the VASP (Vienna Ab initio Simulation Package) software.^11^ The interaction between the ion and valence electrons was described by the PAW (projector-augmented wave) potential.^12^ The GGA-PBE (generalized gradient approximation with Perdew-Burke-Ernzerhof) functional was chosen to describe the exchange and correlation energies of electrons. To ensure the computational accuracy, the energy cutoff for plane-wave was set to 400 eV, and the convergency criteria for energy and max force were set to 1×10^-5^ eV/atom and 0.03 eV/Å. The surface of Fe-N_4_/C with a 6×6 supercell was constructed to simulate the reaction mechanism for toluene oxidation process. A 20 Å vacuum region was adopted for all slab structures to avoid interaction with the adjacent unit in the Z-direction. The Monkhorst-Pack method was used to sample the Brillouin zone with the k-points of 2×2×1.^13^ The climbing image nudged elastic band (CINEB) method was selected for the transition states (TS) search and activation energies determination with the convergence criterion of 0.05 eV/Å.^14^

The adsorption Gibbs free energies (*G*_ads_) for key species (X) during reactions were calculated through Eq. 1, where the *G*_X/slab_, *G*_X_, *G*_slab_ represent the Gibbs free energy of the adsorbed system, isolated species in the gas phase, and pure surface, respectively. All energies were corrected with zero-point energy. The Gibbs free energy barriers (Δ*G*_a_) and reaction Gibbs free energies (Δ*G*_r_) of the elementary steps are calculated using Eq. 2, where *G*_TS_, *G*_IS_, *G*_FS_ is the Gibbs free energy of the optimized structure of initial (IS), transition (TS) and final (FS) states, respectively. All Gibbs free energy corrections are done by the VASPKIT mode.^15^

*G*_ads_ = *G*_X/slab_ – *G*_slab_ – *G*_X_ Eq. 1

$\text{∆G}_{\text{a}}^{\text{≠}}\text{ = }\text{G}_{\text{TS}} \text{- }\text{G}_{\text{IS}}\text{; and }{\text{∆}\text{G}}_{\text{r}}\text{ = }\text{G}_{\text{FS}} \text{- }\text{G}_{\text{IS}}$ Eq. 2

**
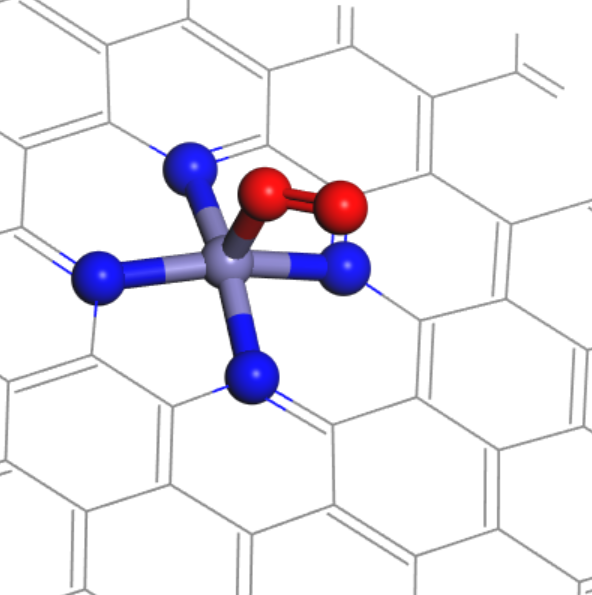
**

**Fig. S14.** Model of O_2_ adsorption on Fe@NC-800, Eads_O_2_ (-0.17 eV).

**S6. Possible reaction pathway for oxidative esterification of alkylarenes**

**Fig. S15.** Possible reaction pathway for Fe@NC-800 catalyzed oxidative CH-esterification of alkylarenes via C-C bond cleavage.

**S7. NMR data**

**Methyl 1-Naphthoate: ^1^H NMR (500 MHz, DMSO-d_6_)** δ 8.81 – 8.74 (m, 1H), 8.21 – 8.10 (m, 2H), 8.05 – 7.98 (m, 1H), 7.66 (ddt, *J* = 8.5, 6.7, 2.0 Hz, 1H), 7.59 (qdd, *J* = 8.7, 4.6, 1.7 Hz, 2H), 3.96 – 3.92 (m, 3H).

**^13^C NMR (126 MHz, DMSO-d_6_)** δ 167.8, 133.9, 133.8, 130.9, 130.4, 129.2, 128.3, 127.2, 126.8, 125.6, 125.3, 52.6.

**Methyl 2-Naphthoate: ^1^H NMR (500 MHz, CDCl_3_)** δ 8.63 – 8.59 (s, 1H), 8.06 (s, 1H), 7.95 (d, *J* = 8.1 Hz, 1H), 7.87 (dd, *J* = 8.5, 2.2 Hz, 2H), 7.56 (dddd, *J* = 23.9, 8.1, 6.8, 1.4 Hz, 2H), 3.98 (s, 3H).

**^13^C NMR (126 MHz, CDCl_3_)** δ 167.3, 135.5, 132.5, 131.1, 129.4, 128.2, 128.2, 127.8, 127.4, 126.6, 125.2, 52.2.

**Methyl 4-methylbenzoate: ^1^H NMR (300 MHz, CDCl_3_)** δ 7.93 (dt, *J* = 8.0, 0.4 Hz, 2H), 7.26 – 7.21 (m, 2H), 3.90 (s, 3H), 2.40 (s, 3H).

**^13^C NMR (75 MHz, CDCl_3_)** δ 167.2, 143.6, 129.6, 129.1, 127.4, 51.9, 21.6.

**Methyl 4-(tert-butyl)benzoate: ^1^H NMR (300 MHz, CDCl_3_)** δ 8.01 – 7.93 (m, 2H), 7.48 – 7.41 (m, 2H), 3.90 (s, 3H), 1.34 (s, 9H).

**^13^C NMR (75 MHz, CDCl_3_)** δ 167.2, 156.5, 129.5, 127.4, 125.3, 51.9, 35.1, 31.1.

**Methyl 4-phenylbenzoate: ^1^H NMR (500 MHz, CDCl_3_)** δ 8.13 – 8.07 (m, 2H), 7.68 – 7.59 (m, 4H), 7.50 – 7.43 (m, 2H), 7.41 – 7.36 (m, 1H), 3.93 (s, 3H).

**^13^C NMR (126 MHz, CDCl_3_)** δ 167.0, 145.6, 140.0, 130.1, 128.9, 128.9, 128.1, 127.3, 127.0, 52.1.

**Methyl 2-fluorobenzoate: ^1^H NMR (300 MHz, CDCl_3_)** δ 7.91 (dddd, *J* = 7.7, 7.3, 1.9, 0.4 Hz, 1H), 7.53 – 7.44 (m, 1H), 7.21 – 7.06 (m, 2H), 3.90 (s, 3H).

**^13^C NMR (75 MHz, CDCl_3_)** δ 164.8, 163.6, 160.2, 134.5, 134.4, 132.1, 124.0, 123.9, 118.7, 118.5, 117.1, 116.8, 52.3.

**Methyl 4-chlorobenzoate: ^1^H NMR (300 MHz, CDCl_3_)** δ 8.00 – 7.95 (m, 2H), 7.44 – 7.38 (m, 2H), 3.92 (s, 3H).

**^13^C NMR (75 MHz, CDCl_3_)** δ 166.3, 139.4, 131.0, 128.7, 128.6, 52.3.

**Methyl 4-bromobenzoate: ^1^H NMR (300 MHz, DMSO-d_6_)** δ 7.91 – 7.84 (m, 2H), 7.77 – 7.71 (m, 2H), 3.86 (s, 3H).

**^13^C NMR (75 MHz, DMSO-d_6_)** δ 166.0, 132.4, 131.6, 129.3, 127.8, 52.8.

**Methyl 4-iodobenzoate: ^1^H NMR (500 MHz, CDCl_3_)** δ 7.82 – 7.78 (m, 2H), 7.74 (dd, *J* = 8.6, 1.7 Hz, 2H), 3.91 (d, *J* = 1.1 Hz, 3H).

**^13^C NMR (126 MHz, CDCl_3_)** δ 166.6, 137.7, 131.0, 129.6, 100.7, 52.3.

**Methyl 4-(trifluoromethyl)benzoate: ^1^H NMR (500 MHz, CDCl_3_)** δ 8.15 (dp, *J* = 7.7, 0.9 Hz, 2H), 7.73 – 7.67 (m, 2H), 3.96 (s, 3H).

**^13^C NMR (126 MHz, CDCl_3_)** δ 165.9, 134.9, 134.6, 134.4, 134.1, 133.5, 133.5, 133.4, 133.4, 130.1, 127.0, 125.5, 125.5, 125.4, 124.8, 122.6, 120.5, 52.6.

**Methyl 3,4-difluorobenzoate: ^1^H NMR (500 MHz, CDCl_3_)** δ 7.89 – 7.78 (m, 2H), 7.26 – 7.17 (m, 1H), 3.92 (s, 3H).

**^13^C NMR (126 MHz, CDCl_3_)** δ 165.2, 154.7, 154.6, 152.6, 152.5, 151.1, 151.0, 149.1, 149.0, 127.2, 127.1, 126.6, 126.5, 119.0, 118.9, 117.4, 117.3, 52.5.

**Methyl 3,4-dichlorobenzoate: ^1^H NMR (300 MHz, DMSO-d_6_)** δ 8.08 (dd, *J* = 2.0, 0.4 Hz, 1H), 7.90 (dd, *J* = 8.4, 2.0 Hz, 1H), 7.81 (dd, *J* = 8.5, 0.3 Hz, 1H), 3.88 (s, 3H).

**^13^C NMR (75 MHz, DMSO-d_6_)** δ 164.9, 136.8, 132.2, 131.7, 131.3, 130.6, 129.6, 53.2.

**Methyl 4-bromo-3-methoxybenzoate: ^1^H NMR (500 MHz, CDCl_3_)** δ 7.60 (d, *J* = 8.1 Hz, 1H), 7.54 (d, *J* = 1.9 Hz, 1H), 7.50 (dd, *J* = 8.1, 1.9 Hz, 1H), 3.95 (s, 3H), 3.92 (s, 3H).

**^13^C NMR (126 MHz, CDCl_3_)** δ 166.4, 155.9, 133.3, 130.6, 122.9, 117.5, 112.4, 56.4, 52.4.

**Methyl 3-iodo-4-methoxybenzoate: ^1^H NMR (500 MHz, CDCl_3_)** δ 8.45 (dq, *J* = 4.6, 2.5 Hz, 1H), 8.01 (dtd, *J* = 7.8, 5.3, 2.9 Hz, 1H), 6.82 (ddd, *J* = 9.9, 4.9, 3.0 Hz, 1H), 3.93 (dd, *J* = 4.3, 2.4 Hz, 3H), 3.90 – 3.85 (m, 3H).

**^13^C NMR (126 MHz, CDCl_3_)** δ 165.5, 161.6, 141.0, 131.6, 124.3, 110.0, 85.3, 56.6, 52.1.

**Methyl 4-methoxybenzoate: ^1^H NMR (300 MHz, CDCl_3_)** δ 8.04 – 7.94 (m, 2H), 6.96 – 6.86 (m, 2H), 3.88 (s, 3H), 3.85 (s, 3H).

**^13^C NMR (75 MHz, CDCl_3_)** δ 166.9, 163.3, 131.6, 122.6, 113.6, 55.4, 51.9.

**Methyl 3-methoxy-2-naphthoate: ^1^H NMR (500 MHz, CDCl_3_)** δ 8.30 (s, 1H), 7.81 (d, *J* = 8.2 Hz, 1H), 7.73 (d, *J* = 8.2 Hz, 1H), 7.51 (t, *J* = 7.6 Hz, 1H), 7.37 (t, *J* = 7.5 Hz, 1H), 7.20 (s, 1H), 3.99 (s, 3H), 3.95 (s, 3H).

**^13^C NMR (126 MHz, CDCl_3_)** δ 166.7, 155.7, 136.1, 132.8, 128.7, 128.4, 127.5, 126.5, 124.4, 121.7, 106.8, 56.0, 52.3.

**Methyl 3-phenoxybenzoate: ^1^H NMR (500 MHz, CDCl_3_)** δ 7.81 (dt, *J* = 7.7, 1.3 Hz, 1H), 7.70 (dd, *J* = 2.5, 1.6 Hz, 1H), 7.45 – 7.36 (m, 3H), 7.24 (ddd, *J* = 8.1, 2.6, 1.0 Hz, 1H), 7.16 (tt, *J* = 7.4, 1.1 Hz, 1H), 7.07 – 7.02 (m, 2H), 3.92 (s, 3H).

**^13^C NMR (126 MHz, CDCl_3_)** δ 166.5, 157.5, 156.7, 132.0, 129.9, 129.8, 124.3, 123.8, 123.3, 119.6, 119.1, 52.2.

**Methyl 3,4-dimethoxybenzoate: ^1^H NMR (300 MHz, DMSO-d_6_)** δ 7.60 (dd, *J* = 8.4, 2.0 Hz, 1H), 7.45 (d, *J* = 2.1 Hz, 1H), 7.08 (d, *J* = 8.5 Hz, 1H), 3.85 – 3.80 (m, 9H).

**^13^C NMR (75 MHz, DMSO-d_6_)** δ 166.5, 153.4, 148.9, 123.6, 122.2, 112.1, 111.6, 56.2, 56.0, 52.4.

**Methyl 3,5-dimethoxybenzoate:** **^1^H NMR (500 MHz, CDCl_3_)** δ 7.18 (d, *J* = 2.4 Hz, 2H), 6.64 (t, *J* = 2.4 Hz, 1H), 3.90 (s, 3H), 3.82 (s, 6H).

**^13^C NMR (126 MHz, CDCl_3_)** δ 166.9, 160.7, 132.0, 107.1, 105.8, 55.6, 52.2.

**Methyl 3,4,5-trimethoxybenzoate: ^1^H NMR (500 MHz, DMSO-d_6_)** δ 7.33 – 7.10 (m, 2H), 3.85 (s, 3H), 3.84 (s, 6H), 3.75 (s, 3H).

**^13^C NMR (126 MHz, DMSO-d_6_)** δ 166.3, 153.2, 142.2, 125.2, 106.9, 60.6, 56.5, 52.7.

**Methyl 4-nitrobenzoate: ^1^H NMR (500 MHz, DMSO-d_6_)** δ 8.38 – 8.28 (m, 1H), 8.23 – 8.12 (m, 1H), 3.92 (dd, *J* = 5.1, 2.5 Hz, 1H).

**^13^C NMR (126 MHz, DMSO-d_6_)** δ 165.2, 150.6, 135.4, 131.0, 124.3, 53.3.

**Methyl picolinate: ^1^H NMR (300 MHz, CDCl_3_)** δ 8.72 (ddd, *J* = 4.8, 1.8, 0.9 Hz, 1H), 8.11 (dt, *J* = 7.9, 1.0 Hz, 1H), 7.83 (td, *J* = 7.7, 1.7 Hz, 1H), 7.46 (ddd, *J* = 7.6, 4.7, 1.2 Hz, 1H), 3.99 (s, 3H). **^13^C NMR (75 MHz, CDCl_3_)** δ 165.7, 149.8, 147.9, 137.1, 127.0, 125.1, 52.9.

**Methyl 2-pyrazinecarboxylate: ^1^H NMR (300 MHz, DMSO-d_6_)** δ 9.21 (dd, *J* = 1.5, 0.4 Hz, 1H), 8.91 (dd, *J* = 2.5, 0.4 Hz, 1H), 8.83 (dd, *J* = 2.5, 1.5 Hz, 1H), 3.93 (s, 3H).

**^13^C NMR (75 MHz, DMSO-d_6_)** δ 164.6, 148.6, 146.0, 145.3, 143.4, 53.2.

**Methyl quinoline-2-carboxylate: ^1^H NMR (500 MHz, DMSO-d_6_)** δ 8.61 – 8.52 (m, 1H), 8.20 – 8.03 (m, 3H), 7.93 – 7.84 (m, 1H), 7.79 – 7.70 (m, 1H), 3.97 (s, 3H).

**^13^C NMR (126 MHz, DMSO-d_6_)** δ 165.8, 148.1, 147.3, 138.1, 131.1, 130.3, 129.3, 129.2, 128.5, 121.2, 53.1.

**Methyl 6-bromoquinoline-2-carboxylate: ^1^H NMR (500 MHz, CDCl_3_)** δ 8.11 (s, 2H), 8.06 (d, *J* = 9.0 Hz, 1H), 7.93 (d, *J* = 2.2 Hz, 1H), 7.75 (dd, *J* = 9.0, 2.2 Hz, 1H), 4.00 (s, 3H).

**^13^C NMR (126 MHz, CDCl_3_)**, δ 165.6, 148.2, 146.0, 136.3, 133.9, 132.2, 130.2, 129.6, 123.0, 121.9, 53.3.

**Methyl isoquinoline-3-carboxylate: ^1^H NMR (500 MHz, CDCl_3_)** δ 9.34 (d, *J* = 1.2 Hz, 1H), 8.61 (d, *J* = 1.0 Hz, 1H), 8.10 – 8.04 (m, 1H), 8.02 – 7.96 (m, 1H), 7.78 (dddd, *J* = 20.6, 8.2, 7.0, 1.3 Hz, 2H), 4.07 (s, 3H).

**^13^C NMR (126 MHz, CDCl_3_)** δ 165.2, 154.8, 152.7, 151.2, 149.1, 127.3, 126.6, 119.1, 117.5, 117.3, 52.5.

**Methyl quinoline-6-carboxylate: ^1^H NMR (500 MHz, CDCl_3_)** δ 9.01 (dd, *J* = 4.3, 1.9 Hz, 1H), 8.60 (d, *J* = 2.1 Hz, 1H), 8.29 (ddd, *J* = 18.7, 8.6, 1.9 Hz, 2H), 8.15 (d, *J* = 8.8 Hz, 1H), 7.48 (dd, *J* = 8.4, 4.2 Hz, 1H), 4.00 (s, 3H).

**^13^C NMR (126 MHz, CDCl_3_)** δ 166.6, 152.5, 150.1, 137.3, 131.0, 129.0, 128.2, 127.4, 121.9, 52.4.

**Methyl indole-5-carboxylate: ^1^H NMR (500 MHz, CDCl_3_)** δ 8.64 (s, 1H), 8.46 – 8.41 (m, 1H), 7.90 (dd, *J* = 8.7, 1.7 Hz, 1H), 7.38 (dt, *J* = 8.6, 0.8 Hz, 1H), 7.27 – 7.23 (m, 1H), 6.63 (ddd, *J* = 3.1, 2.0, 0.9 Hz, 1H), 3.93 (s, 3H).

**^13^C NMR (126 MHz, CDCl_3_)** δ 168.4, 138.5, 127.5, 125.7, 123.8, 123.3, 121.8, 110.8, 103.9, 51.9.

**Methyl indole-4-carboxylate: ^1^H NMR (300 MHz, CDCl_3_)** δ 8.47 (s, 1H), 7.99 (dd, *J* = 7.5, 1.0 Hz, 1H), 7.67 (dt, *J* = 8.1, 1.0 Hz, 1H), 7.42 (dd, *J* = 3.2, 2.5 Hz, 1H), 7.34 – 7.30 (m, 1H), 7.28 (d, *J* = 4.7 Hz, 1H), 4.06 (s, 3H).

**^13^C NMR (75 MHz, CDCl_3_)** δ 168.1, 136.6, 127.4, 126.2, 123.5, 121.6, 121.2, 115.9, 104.0, 51.8.

**Methyl 1H-benzo[d]imidazole-2-carboxylate: ^1^H NMR (500 MHz, DMSO-d_6_)** δ 13.51 (s, 1H), 7.68 (s, 2H), 7.33 (dd, *J* = 6.0, 3.1 Hz, 2H).

**Methyl benzooxazole-2-carboxylate: ^1^H NMR (500 MHz, DMSO-d_6_)** δ 7.98 – 7.83 (m, 2H), 7.66 – 7.47 (m, 2H), 3.99 (s, 3H).

**^13^C NMR (126 MHz, DMSO-d_6_)** δ 156.8, 153.0, 150.6, 140.5, 128.7, 126.3, 122.1, 112.4, 53.9.

**S8. NMR spectra**

**S9. References**

1. Z. Klencsar, E. Kuzmann, A. Vertes, “User-friendly software for Mossbauer spectrum analysis,” *Journal of Radioanalytical and Nuclear Chemistry* **210** (1996): 105-118.

2. L. Kesavan, R. Tiruvalam, M. H. A. Rahim, et al., “Solvent-Free Oxidation of Primary Carbon-Hydrogen Bonds in Toluene Using Au-Pd Alloy Nanoparticles,” *Science* **331** (2011): 195-199.

3. D. Khalili, M. Rousta, A. Khalafi-Nezhad, E. Ebrahimi, “From methylarenes to esters: efficient oxidative Csp^3^–H activation promoted by CuO decorated magnetic reduced graphene oxide,” *New Journal of Chemistry* **46** (2022): 14052-14064.

4. D. S. Mannel, et al., “Discovery of Multicomponent Heterogeneous Catalysts via Admixture Screening: PdBiTe Catalysts for Aerobic Oxidative Esterification of Primary Alcohols,” *Journal of the American Chemical Society* **139** (2017): 1690-1698.

5. H. Su, et al., “Activating Cobalt Nanoparticles via the Mott−Schottky Effect in Nitrogen-Rich Carbon Shells for Base-Free Aerobic Oxidation of Alcohols to Esters,” *Journal of the American Chemical Society* **139** (2017): 811-818.

6. R. V. Jagadeesh, et al., “Selective Oxidation of Alcohols to Esters Using Heterogeneous Co_3_O_4_-N@C Catalysts under Mild Conditions,” *Journal of the American Chemical Society* **135** (2013): 10776-10782.

7. W. Zhong, et al., “Base-Free Oxidation of Alcohols to Esters at Room Temperature and Atmospheric Conditions using Nanoscale Co-Based Catalysts,” *ACS Catalysis* **5** (2015): 1850-1856.

8. M. Liu, et al., “Transformation of alcohols to esters promoted by hydrogen bonds using oxygen as the oxidant under metal-free conditions” *Science Advances* **4**, (2018): eaas9319.

9. K. Suzuki, et al., “Aerobic Oxidative Esterification of Aldehydes with Alcohols by Gold−Nickel Oxide Nanoparticle Catalysts with a Core−Shell Structure,” *ACS Catalysis* **3** (2013): 1845-1849.

10. R. L. Oliveira, et al., “Clean preparation of methyl esters in one-step oxidative esterification of primary alcohols catalyzed by supported gold nanoparticles,” *Green Chemistry* **11** (2009): 1366-1370.

11. G. Kresse, J. Furthmüller, “Efficient iterative schemes for ab initio total-energy calculations using a plane-wave basis set,” *Physical Review B* **54** (1996): 11169-11186.

12. P. E. Blöchl, “Projector augmented-wave method,” *Physical Review B* **50** (1994): 17953-17979.

13. J. D. Pack, H. J. Monkhorst, "Special points for Brillouin-zone integrations—a reply,” *Physical Review B* **16** (1977): 1748-1749.

14. G. Henkelman, B. P. Uberuaga, H. Jonsson, “A climbing image nudged elastic band method for finding saddle points and minimum energy paths,” *The Journal of Chemical Physics* **113** (2000): 9901-9904.

15. V. Wang, N. Xu, J.-C. Liu, et al., “VASPKIT: A user-friendly interface facilitating high-throughput computing and analysis using VASP code,” *Computer Physics Communications* **267** (2021): 108033.
